# Diversity and Dissemination of Methicillin-Resistant *Staphylococcus aureus* (MRSA) Genotypes in Southeast Asia

**DOI:** 10.3390/tropicalmed7120438

**Published:** 2022-12-13

**Authors:** Nurul Amirah Mohamad Farook, Silvia Argimón, Muttaqillah Najihan Abdul Samat, Sharifah Azura Salleh, Sunita Sulaiman, Toh Leong Tan, Petrick Periyasamy, Chee Lan Lau, Zalina Ismail, Nor Azila Muhammad Azami, Mia Yang Ang, Hui-min Neoh

**Affiliations:** 1 UKM Medical Molecular Biology Research Institute (UMBI), Universiti Kebangsaan Malaysia, Kuala Lumpur 56000, Malaysia; 2 Centre for Genomic Pathogen Surveillance, Big Data Institute, University of Oxford, Oxford OX3 7LE, UK; 3 Department of Medical Microbiology and Immunology, Faculty of Medicine, Universiti Kebangsaan Malaysia, Kuala Lumpur 56000, Malaysia; 4 Infection Control Unit, Hospital Canselor Tuanku Muhriz, Universiti Kebangsaan Malaysia, Kuala Lumpur 56000, Malaysia; 5 Department of Emergency Medicine, Faculty of Medicine, Universiti Kebangsaan Malaysia, Kuala Lumpur 56000, Malaysia; 6 Department of Medicine, Faculty of Medicine, Universiti Kebangsaan Malaysia, Kuala Lumpur 56000, Malaysia; 7 Department of Pharmacy, Universiti Kebangsaan Malaysia, Kuala Lumpur 56000, Malaysia; 8 Department of Gene Diagnostics and Therapeutics, National Center for Global Health and Medicine, Tokyo 162-8655, Japan; 9 Department of Clinical Genome Informatics, Graduate School of Medicine, The University of Tokyo, Tokyo 113-0033, Japan; 10 Faculty of Health Sciences, Universiti Kebangsaan Malaysia, Kuala Lumpur 56000, Malaysia

**Keywords:** MRSA, MRSA genotyping, Southeast Asia

## Abstract

Methicillin-resistant *Staphylococcus aureus* (MRSA) is a successful pathogen that has achieved global dissemination, with high prevalence rates in Southeast Asia. A huge diversity of clones has been reported in this region, with MRSA ST239 being the most successful lineage. Nonetheless, description of MRSA genotypes circulating in the Southeast Asia region has, until now, remained poorly compiled. In this review, we aim to provide a better understanding of the molecular epidemiology and distribution of MRSA clones in 11 Southeast Asian countries: Singapore, Malaysia, Thailand, Vietnam, Cambodia, Lao People’s Democratic Republic (PDR), Myanmar, Philippines, Indonesia, Brunei Darussalam, and Timor-Leste. Notably, while archaic multidrug-resistant hospital-associated (HA) MRSAs, such as the ST239-III and ST241-III, were prominent in the region during earlier observations, these were then largely replaced by the more antibiotic-susceptible community-acquired (CA) MRSAs, such as ST22-IV and PVL-positive ST30-IV, in recent years after the turn of the century. Nonetheless, reports of livestock-associated (LA) MRSAs remain few in the region.

## 1. Introduction

The Southeast Asia region, consisting of 11 countries (Singapore, Malaysia, Thailand, Vietnam, Cambodia, Lao PDR, Myanmar, Philippines, Indonesia, Brunei Darussalam, and Timor-Leste) is well-known for its pristine beaches and tropical forests. Nevertheless, many cities in the region are densely populated areas that facilitate the spread of multidrug-resistant pathogens [1,2], such as methicillin-resistant *Staphylococcus aureus* (MRSA). MRSA is currently listed as one of the top pathogens in the region, and it is responsible for more than 100,000 deaths, contributing to the global antimicrobial resistance burden [3].

Since acquiring methicillin resistance, MRSAs have expanded widely across the globe in many hospitals, causing a variety of nosocomial infections, such as pneumonia, bacteraemia, and surgical-site infections via the carriage of virulence factors [4]. The pathogen carries SCC*mec*, a cassette of genes that encodes the methicillin-resistance conferring *mec*A gene and cassette chromosome recombinases (*ccr*). Different combinations of *mec* and *ccr* types in MRSAs give rise to the SCC*mec* genotype, where 13 distinct types of SCC*mec* elements (type I to XIII) have been described to date in global MRSAs [5]. This pathogen is also commonly characterized using the multi-locus sequence type (MLST) scheme, where lineages of MRSAs are categorised into sequence types (STs) based upon sequences in seven *S. aureus* housekeeping genes [6]. STs that share at least five out of seven alleles are further grouped into clonal complexes (CC) [7].

MRSA clones were first described in hospital patients and nosocomial in aetiology. These are now designated as “hospital-acquired” MRSAs (HA-MRSAs), where global HA-MRSA clones include CC5-SCC*mec*II (USA100), CC5-SCC*mec*IV (USA800), CC8-SCCmecIV (USA500), CC22-SCCmecIV (EMRSA-15), CC30-SCC*mec*II (EMRSA-16), CC45-SCC*mec*IV, and ST239-SCC*mec*III [8] harboring SCC*mec* elements of types I, II, or III [5]. In the 1980s, reports of MRSA infections in the community (community-acquired MRSAs, CA-MRSAs) among healthy people and from patients within 48 to 72 h of hospital admission (with no medical history of MRSA infection or colonization and hospitalization) [9] began to surface [10]. Interestingly, CA-MRSA clones are mostly genotypically different from HA-MRSAs, where dominant clones include ST8-IV (USA300), ST1-IV (WA-1, USA400), ST30-IV (South West Pacific clone), ST59-V (Taiwan clone), and ST80-IV (European clone) [11]. Early CA-MRSA clones were also reported to carry the Panton–Valentine leukocidin (PVL) genes (*lukS-PV* and *lukF-PV*), though they were later found not to be characteristic of all CA-MRSAs [12,13]. More recently, MRSAs isolated from livestock (livestock-associated MRSA, LA-MRSA) with potential dissemination in humans have been reported [14], where prominent LA-MRSAs are mostly CC398 (including ST398, ST752, or ST753) [15].

Successful MRSA lineages have achieved global dissemination, where Asia records one of the highest prevalence in the world [16]. In countries such as Singapore, Malaysia, Philippines and Thailand, national-level antimicrobial resistance surveillance programs have been implemented to track the resistance trend of MRSA [17]. By contrast, diagnostic and antibiotic-susceptibility testing capacities in diagnostic laboratories remain limited in countries, such as Myanmar, Laos and Cambodia [18,19]. As there is no standardized surveillance protocol in Southeast Asia, it is difficult to determine the epidemiology of MRSA isolates in the region [19,20].

This review compiles results from MRSA epidemiological studies carried out in diagnostic laboratories and research institutes in Southeast Asian countries since the 1970s to provide a better understanding of the dissemination of MRSA clones in this region, together with their antibiotic resistance profile, antibiotic resistance gene determinants, and virulence factors. Overall, prevalence rates of MRSA differ widely throughout regions and countries. A list of historical and current MRSA clones circulating in Southeast Asian countries is shown in Table 1. The prevalence of MRSA infections in Southeast Asia ranged between 20% and 30% [20], and ST239-SCC*mec* III has been frequently reported as the predominant lineage circulating in many countries in this region.

## 2. Molecular Epidemiology and Distribution MRSA Clones

### 2.1. Singapore

The first MRSA strains in Singapore were reported in the 1970s [21]. Nevertheless, molecular profiling of MRSAs in Singapore only began later in the mid-1980s where the dominant MRSA population was identified as ST239-III, the oldest pandemic MRSA clone well-known with its multidrug-resistant profile [22,23]. Analysis of MRSA historical isolates from the 1980s and 1990s from Singapore General Hospital (SGH) demonstrated evidence of ST239 predominance [24,25], and in 2003, the first ST22-IV clone was discovered, where the ST22-IV clone was highly susceptible to several non–beta-lactam antibiotics compared to ST239-III. Only 33.5% were identified as ST22-IV [26], and the subsequent surveillance data revealed an increasing prevalence of ST22-IV ranging between 25% to 66.1%. This clone would later successfully establish itself in most Singaporean hospitals [25,27]. Later, genome sequencing of a large number of MRSAs from three general hospitals (SGH, Tan Tock Seng Hospital, and Changi General Hospital) isolated between 2000 to 2010 demonstrated that ST239 MRSA was often transferred within the healthcare setting and was introduced multiple times into the hospitals [25]. Later in 2013, a high proportion of ST22 MRSAs were found to exhibit high levels of mupirocin resistance, an antibiotic exclusively introduced in 1996 for MRSA decolonization [28]. In addition to the gradual replacement of ST239 with ST22, another major global MRSA lineage, ST45, was also soon found to be overtaking ST239 in prevalence [28], though the true dynamics of the ST45 spread could not be established yet. ST45 lineage is widely distributed throughout Africa, Australia, Asia, Europe, and North America [29].

Early reports of community-associated MRSA (CA-MRSA) in Singapore showed that these strains were only found sporadically. A study of MRSA isolates from SGH that fit the criteria for possible CA-MRSA, conducted from January 2001 to April 2004, revealed the presence of international CA-MRSA clones, such as the European clone (ST80-IV), Southwest Pacific clone (ST30-IV), Taiwanese clone (ST59-V), and a few other CA-MRSAs that harbored SCC*mec* type V (ST1-V and ST524-V) [30]. Following this, SGH and a few other public hospitals have conducted continuous surveillance to monitor CA-MRSA infection rates, where another six PVL-positive CA-MRSA cases were discovered between April and October 2004: four of the isolates were ST30 and the rest were ST59 [31]. CA-MRSA clones were suggested to be gradually establishing themselves in Singapore, and it was predicted that this successful CA-MRSA clone (ST30-IV) might become the dominant CA-MRSA clone in Singapore. This prediction was proven in another study conducted from May 2004 to June 2005 involving 37 CA-MRSA isolates of five different hospitals, where ST30-SCC*mec* type IVc was identified as the predominant clone [32].

Subsequently, the first CA-MRSA outbreak was reported in Singapore in 2010. An outbreak of 27 skin and soft tissue infections in an international school of 3800 students was involved where 8 out of 27 cases were due to ST8 [33]. Most cases presented with identical patterns of resistance to ciprofloxacin and erythromycin, where the isolates were resistant to no more than three types of antibiotics. ST8 (USA300) has been linked to outbreaks among sports teams, prisoners, military personnel, and childcare centers in many regions of the world [34]. Most of the students involved in the cases travelled to the US during their summer break and might have carried the CA-MRSA strain back to Singapore when they returned [13].

By contrast, the presence of LA-MRSA lineage ST398 SCC*mec* type V has also been documented in Singapore [35]. These ST398 were isolated from pigs and displayed resistance to ciprofloxacin, clindamycin, and tetracycline. The clone possessed the γ-haemolysin toxin gene. Interestingly, the predominant clone circulating in Singapore at the same period (ST22-IV) was also discovered in a pig imported from Indonesia (for research) and its attending scientist [35].

### 2.2. Malaysia

MRSA has been isolated in Malaysia since the mid-1970s [36]. The earliest known study of MRSA in Malaysia dates back to 1984 at the special care nursery of Kuala Lumpur Maternity Hospital, where 53 out of 858 babies were found to be colonized or infected by multidrug-resistant MRSA [37]. Later, in 1990, the prevalence of MRSA seemed to be growing steadily not only in nursery and pediatric wards but also in the orthopedics, surgical, and urology wards [36]. It is very difficult to uncover the dominant MRSA population before the 2000s as no molecular information has been published about these isolates. 

The following decade, a significant number of studies began investigating the molecular profiles of Malaysian MRSAs isolated from the Klang Valley (central region in Peninsular Malaysia with the densest population) and identified ST239-III predominance in the country [38,39,40]. In 2006, Neela et al. reported ST239-III or IIIa with spa t037 as the most dominant clone circulating at one public hospital in the Klang Valley [41]. The same team continued to investigate MRSA epidemiology in one of Malaysia’s largest public hospitals (Hospital Kuala Lumpur) in late 2007, where the majority of the MRSA isolates also belonged to one MRSA clone, supporting the evidence of ST239 predominance [40]. ST239 MRSA demonstrated a high resistance to beta-lactam antibiotics and was very persistent within hospital settings, where Lim et al. reported it as the dominant clone in two different studies conducted in 2003 and 2008. [38].

During the same period, the presence of a few PVL-positive CA-MRSA clones, such as ST30-IV, ST1-V and ST188-V, were identified [40,42]. A few recent studies also reported CA-MRSA clones harboring SCC*mec* type V and the PVL gene; however, due to the limited number of studies, the true prevalence of this clone remains concealed. While ST239-III continued its dominance in the country, around 2010 the growing proportion of ST22-IV (EMRSA-15) in Malaysian hospitals hinted that the MRSA epidemiology in Malaysia might be changing, just like neighboring Singapore. This highly transmissible HA-MRSA clone was first reported in the country by Lim et al. in 2007, and subsequent studies over the next few years continued to report its prevalence in Malaysian hospitals [38,42,43,44]. It was observed that these SCC*mec* type IV clones consistently exhibited high resistance toward ciprofloxacin and susceptibility to four or more non–beta-lactam antibiotics [38]. Interestingly, Ghaznavi-Rad et al. reported that the ST22 carried higher superantigenic enterotoxin genes (*seg*, *sei*, *sem*, *sen*, *seo)*, whereas ST239-III mostly harbored only *sea* [40]. Indeed, a recent report from 2016 to 2017 of MRSAs isolated from Kelantan, a state located at the north of Peninsular Malaysia just across the border from Thailand, revealed that majority of MRSA isolates were ST22 (68.2%), followed by ST239 (29.5%) [45]. Another molecular surveillance in a teaching hospital, Hospital Canselor Tuanku Muhriz (HCTM), conducted in 2017, reported clonal replacement of the previous dominant MRSA clone of SCC*mec* type III-SCC*mercury* (these clones were also found to carry *sea+cna*) with SCC*mec* type IV (which carried *seg + sei*) after an eight-year interval from the first molecular surveillance carried out in the hospital in 2009 [44,46]. This clonal replacement phenomenon has been observed in many countries, such as Singapore, the United States, and Germany [25,47,48]. Characterization of MRSA bacteraemia in another hospital located in the Klang Valley in 2011 to 2013, revealed diversity of SCC*mec* type IV MRSA clones of various STs (ST1, ST5, ST6, ST22, and ST1137), with resistance toward erythromycin, ciprofloxacin, clindamycin, and gentamicin. [43,49]. At that point, it seemed that most of the MRSAs circulating in Malaysia were SCC*mec* type IV. 

Meanwhile, the prevalence of MRSA in livestock (pigs and poultry) and their handlers were reportedly low in the country [50,51,52]. Neela et al. identified the presence of multi-resistant ST9 and ST1 among pigs and pig handlers instead of the traditional LA-MRSA clone, ST398 [50]. Both STs carried SCC*mec* type V, bearing enterotoxin genes (*seg*, *seb*, *see*), fibronectin-binding protein (*fnb*) and collagen (*cna;* present only in ST1). A recent report on LA-MRSAs isolated from dairy cattle in Kelantan, a state located at the Thai-Malaysian border, reported a 17.89% prevalence and presence of *mec*C in bovine milk and cattle nasal swabs [52]. 

### 2.3. Thailand

The presence of MRSA in Thailand was recorded as early as 1988 from four patients from Siriraj hospital in central Thailand [53]. A year later, another MRSA case was also discovered from surgical wound infections in the gynaecology ward at another hospital in the same region [54]. From 1990 to 1991, an outbreak of MRSA occurred in the burn unit of Siriraj hospital, leading to a temporary ward closure due to difficulties in eradicating the outbreak [55]. According to the available literature, no further studies have been conducted to identify the MRSA clones that circulated the country in the 1990s except for a multinational surveillance by Chongtrakool et al., where a collection of MRSAs from 1998 to 1999 from central Thailand revealed the dominance of MRSA ST239 with SCC*mec* type III [22]. MRSA ST239 was still the prevailing clone in central Thailand five years later, as shown by surveillance conducted between 2004 to 2006, which also reported a HA-MRSA rate of 57% (180/316) with resistance to at least five types of antibiotics [22,56]. Aside from ST239-III, the presence of another pandemic MRSA lineage, ST5-II (New York /Japan clone) was also detected from early Srinagarind Hospital MRSAs between 2002 and 2003 [57,58]. Following this period, the majority of the Thai MRSA studies continued to document the predominance of ST239-III in Thailand, especially in MRSA isolates from hospitals located in the north eastern region, such as Srinagarind Hospital and Sappasithiprasong Hospital [57,59,60,61].

The first characterization of MRSA isolates in Thailand using whole genome sequencing (WGS) came from a prospective MRSA carriage study conducted in 2008 over 3 months in two intensive care units in the same hospital, which revealed a diverse MRSA ST239 population within the Asian clade [61]. The second WGS study of MRSA isolates recovered from the Thammasat University Hospital in central Thailand from 2012 to 2015 revealed the prevalence of ST764-II [62]. Interestingly, this ST belongs to the global ST5 lineage and was frequently found circulating mainly in Japan [63]. 

While ST239-III continued to dominate in certain Thai regions in 2015–2016 [64], SCC*mec* type IV was first noted in this same period from Hua Hin Hospital, a general hospital in western Thailand. Santimaleeworagun et al. reported this strain to be the first CA-MRSA of rare MRSA lineage ST2885, isolated from hospitalized patients, and without the PVL gene [65]. This clone was resistant to beta-lactam antibiotics but susceptible to tetracyclines, clindamycin, and trimethoprim-sulfamethoxazole, making it similar to typical CA-MRSA clones in an antibiogram profile. The prevalence of CA-MRSA cases in Thailand seemed to be low. Among 186 MRSAs isolated from Siriraj Hospital in 2005, only three isolates were identified as suspected cases of CA-MRSA infection [66]. These strains were isolated in two patients within 72 h of hospitalization; however, molecular characteristics of these isolates were not fully investigated. From 2004 to 2006, Song et al. reported a low CA-MRSA incidence of 2.5% (3/122), where MLST typing revealed these isolates to be the multidrug-resistant ST239-III [56]. Characterization of MRSA from the outpatients of Srinagarind Hospital in the same year also reported CA-MRSAs of ST239-III and ST9-IX [59]. SCC*mec* type IX was identified previously in an LA-MRSA clone, ST398 from a Thai participant at the 19th International Pig Veterinary Conference in Denmark [67]. ST398 MRSAs were also reported in later years from 2015 to 2017 among workers in the swine industry, where a majority of the isolates were multidrug-resistant and harbored resistant genes that are commonly found in a LA-MRSA clone (chloramphenicol–florfenicol resistance gene; *cfr*) [68]. By contrast, in most Asian countries, ST9 has been linked with the incidence of LA-MRSA in pigs, cattle, and poultry but not in humans [69]. This particular clone from northern Thailand, however, was isolated from a young boy with chronic impetigo with no hospitalization history. The clone was then reported again in 2019, isolated from a 49-year-old man with bacteraemia pneumonia in the same region. The MRSA carried multiple antibiotic resistance genes, including *mecA*, *blaZ*, *aac(69)-aph(2″)*, *aadD*, *ant(6)-Ia*, *lsa(E)*, *dfrG*, *tet(M)*, *fexA,* and *lnu (B)* [59,70]. Indeed, several recent studies revealed the prevalence of ST9-IX among individuals working in pig farms [71,72], signalling the importance of the clone as the new CA-MRSA emerging in the Thai swine industry community.

A few MRSA studies conducted between 2012 to 2017 observed a low MRSA prevalence in central Thailand [73,74]. Due to limited studies, it remains to be determined if Thai MRSA prevalence has been declining or whether the dominant ST239-III clone has been supplanted by another emerging clone as observed in many other countries. The dominant MRSA genotype circulating in Thailand after 2016 remains to be determined.

### 2.4. Vietnam

Profiling studies of MRSAs in Vietnam revealed varied profiles of the bacteria according to different geographical regions: southern, central, and northern, with the majority of studies conducted in the southern and northern regions [75]. Early genotyping data on representative MRSAs from Ho Chi Minh City (southern region) between 1998 and 2003 uncovered the prevalence of ST239-III as well as the presence of ST241-III and ST346-II clones [22,23]. Both ST239-III and ST241-III clones exhibited resistance to approximately seven or eight antibiotics. Between 2004 to 2006, the MRSA isolation rate in Vietnam was significantly higher compared to other Asian countries [56]. This study reported ST239-III and ST59-IV as the predominant MRSA clones circulating in Vietnam. ST59, known as the Taiwan clone, is one of the early epidemic CA-MRSAs that emerged in 1974 in the country [76]. In May 2006, the dominant ST59 CA-MRSA clone (SCC*mec* type V) caused an outbreak of severe skin infections in nine healthy children in Ho Chi Minh City where the clone was discovered to have been transmitted by an asymptomatic healthcare worker during routine vaccination [77]. This ST59 clone harbored several virulence factors (*PV-luk*, *seg*, *seb*, *hla*, *cap8*) but was not multidrug resistant. It was also one of the prevalent CA-MRSA clones circulating among pediatric populations in China and Taiwan in the same period [78,79].

SCC*mec* typing of the MRSA isolates from four tertiary hospitals in the southern region, (Cho Ray Hospital, Gia Dinh People’s Hospital, 175 Military Hospital, and the Thong Nhat Hospital) isolated between 2008 and 2010 belonged to SCC*mec* type III [80]. Among the HA-MRSA isolates, only 13% were SCC*mec* type IV and V. In contrast, later studies conducted between 2011 to 2014 among pediatric patients in another hospital from southern Vietnam reported the low frequency of SCC*mec* type III and the prominence of MRSAs with SCC*mec* type IV, with the presence of ST45-IV, ST88-IV, and ST2808-IV clones that were multidrug-resistant with reduced susceptibilities to ciprofloxacin, erythromycin, gentamicin, and tetracycline [81]. The significant prevalence of SCC*mec* type IV was further supported by a study conducted in 175 General Hospital between 2013 and 2014 [75]. Meanwhile, approximately 29.8% of population in northern Vietnam carry nasal *S. aureus*, with a MRSA prevalence of 7.9% (80/1016) [82]. An investigation of the population structure of both carriage and invasive strains from the same region over the same study period revealed the prevalence of PVL-positive CC59 and other clonal complexes without the PVL genes, such as CC45 and CC188 [83]. These data further strengthened the evidence of ST59 predominance in this country. Between 2011 to 2014, a study reported that the predominance of a new clone, ST5 with SCC*mec* type II, circulating in the northern hospitals among pediatric patients [81]. This clone was resistant to gentamycin, ciprofloxacin, tetracycline, meropenem, and cephalosporins and habored *mecA*, *aacA/aphD*, *ermA/B/C*, and *tetK/M*. Of note, ST5-II or the New York/Japan clone is one of the international pandemic MRSA clones that is responsible for a high number of infections [84].

Concurrently (2011 to 2014), Son et al. further investigated MRSA isolated from hospitalized adult patients in three different regions of central, southern, and northern Vietnam [81]. The study revealed that MRSAs in all three regions were dominated by SCC*mec* type IV followed with type III and II; MLST typing further revealed the presence of ST188-IV/III and ST239-II isolates but not the ST59 clone. A majority of these STs contained genes conferring resistance to antibiotics, such as tetracycline (*tetK*, *tetM*), aminoglycosides (*aacA/aphD*), macrolides, and lincosamide (*ermA*, *ermB*, and *ermC*). Incidentally, the ST188 has been discovered in Singaporean and Malaysian communities earlier on and reported in 2004 and 2008, respectively [31,42]. More recently, Thuy et al. reported high MRSA colonization rates among adult patients in Vietnamese ICUs of 8.6% (72/838, CA-MRSAs) and 16.2% (59/364, HA-MRSAs) [85,86]. This data imply that MRSA may be a growing concern in Vietnam, which needs to be addressed. The high usage of antibiotics and inappropriate antibiotic prescriptions in most Vietnamese hospitals is also particularly worrying [87]. 

### 2.5. Cambodia

While most Asian countries reported an increasing prevalence of MRSA in the 2000s, a small surveillance in the Battambang region in 2002 did not report the presence of MRSA [88]. Cambodia has an microbiology laboratory capacity compared to its neighboring countries, Thailand and Vietnam, and has only recently started to expand their facilities [89,90]. Therefore, the true prevalence of MRSA prior the 2010s might be obscured, other than data from studies performed in northwest Cambodia.

Interestingly, most MRSAs studied in Cambodia were isolated from pediatric patients. The first MRSA infection was identified in the Angkor teaching hospital in Siem Reap, located at north western Cambodia in January 2006 [91] during the same period when the hospital diagnostics laboratory was just recently established. The study was extended from January 2006 to December 2007, and 16 additional cases of pediatric MRSA infections were reported from three different regions in northwest Cambodia. In all the above 17 suspected cases of CA-MRSA infections, eleven children had skin and soft tissue infections, while another six had invasive diseases. While genotyping of 15 MRSAs revealed the isolates to be ST834-IV, the remaining two MRSAs were the PVL-positive ST121-V clone, which was more susceptible to tested antibiotics (trimethoprim/sulphamethoxazole, ciprofloxacin, and rifampicin) compared to ST834. These infections appeared to be sporadic and associated with an endemic carriage of the causative strains. For unknown reasons, the ST834 genotype has been reportedly linked to pediatric patients and was rarely identified except in East and Southeast Asia and Australia [92,93]. By contrast, ST121 is a global hypervirulent clone disseminated primarily in Europe and Africa as well as in Asia [94]. Nickerson et al. conducted a one-month study in the following year to investigate the prevalence of MRSA carriage in children from the same hospital [95], which reported a low carriage rate of MRSA in children (3.5%, 93/2630). Similarly, MRSA prevalence among hospitalized children in another study from the same hospital also appeared low throughout a 10-year surveillance on antibiotic-resistant bacteria from 2007 to 2016 [96]. Despite the low MRSA rate, the majority of the isolates also belonged to the multidrug-resistant ST834, demonstrating the dominance of ST834 in Cambodia. The surveillance also revealed emergence of new CA-MRSA clones that had previously been reported in other countries, such as ST188, ST45, ST121, and ST9 [31,42,83].

### 2.6. Lao People’s Democratic Republic (PDR)

Reports of MRSA infections from Laos have been very few compared to other Southeast Asian countries due to a lack of a diagnostic microbiology and research infrastructure. Indeed, a few studies published in the 2000s reported the absence of MRSA in Laos. A study on community-acquired bacteraemia in hospitalized patients between 2000 and 2004 at the Mahosot Hospital, located in the capital Vientiane City, did not identify MRSA as the causative pathogen [97]. Similarly, another study of bacteraemia from infants from 2000 to 2011 also reported the absence of MRSA [98]. Nevertheless, Phetsouvanh et al. noted that only 11% of Laotians reside in the developed areas of Laos, such as Vientiane City; hence, the findings of these studies may not reflect the true prevalence of MRSA in Laos [97].

MRSA monitoring and mandatory reporting in hospitals has been implemented since 1993, yet surprisingly, no MRSA from patients or healthcare workers were reported until 2001, when the first two MRSA strains of the country were isolated from different hospitals in Vientiane City [99,100]. Since the genotype and phenotype of these strains differed, the isolates most probably originated from different sources. One isolate harbored SCC*mec* type III with a multidrug-resistant profile, whereas the other carried a new SCC*mec* type, which has not been designated at the time of isolation (presumably type IV), with a more susceptible antibiotic profile. The following year, another presence of SCC*mec* type IV with a similar antibiotic profile was reported in the country: three MRSA isolates from one hospital and one isolate from another hospital in the same region [100]. All MRSA isolates carried β-haemolysin and urease virulence genes, while enterotoxin genes (*seb + sed* or *sed* only) were found only in SCC*mec* type IV MRSA. Despite the identification of MRSA strains, documentation of the occurrences was incomplete, making it hard to elucidate the source of the infections. From July 2012 to June 2014, molecular epidemiology studies on *S. aureus* isolated from skin and soft tissue infections in Mahosot Hospital identified seven MRSA isolates [101]. Genotyping of these isolates revealed the existence of the ST239-III, CC59-V, ST2250-IV, ST2885-V, and CC398-V lineages, i.e., circulating MRSA clones in other countries in Southeast Asia [67,68,83], suggesting regional dissemination across borders. Virulence profiling revealed that ST59/952-V- and CC398-V-encoded PVL genes were linked with the associated patient’s history of abscess. The strains harbored *blaZ*, *ermA*, *ermB*, *tetK*, and *cat*. Exfoliative toxin A gene (*etA*) only was present in ST2885-V together with *cap5* and *sec*, while *sea* was present in ST239, similar to other ST239s in the region.

### 2.7. Myanmar

*S. aureus* is reported to be one of the most frequently isolated healthcare-associated pathogens in Myanmar. Nevertheless, genotyping studies on *S. aureus* or MRSA remain limited [102]. A recent study from 2017 to 2019 described the predominance of ST772-V among the PVL-positive MRSA clinical isolates isolated from Yangon, the largest city in Myanmar [103]. This CA-MRSA clone, also known as the Bengal Bay clone, first appeared in the Indian subcontinent and has since rapidly spread to different continents, reaching Asia, Australasia, Africa, the Middle East, and Europe since the 1990s [104]. ST239-III MRSA and ST2507-III, a single-locus, were scarcely represented among the clinical isolates. In addition, another two well-known international clones, USA300 (ST8-IV) and EMRSA-15 (ST22-IV), were also present during the two-year study period of 2017–2019. ST772-V and ST22-IV were found to be resistant to approximately 7 to 11 tested antibiotics and harbored more virulence factors compared to other clones. Interestingly, prior to this, only ST239-III and ST5-IV were circulating in the country in 2007 and 2012 [102,105]. The clinical ST239-III isolated between 2007 to 2008 was multidrug-resistant, harboring more resistance genes, *blaZ*, *tetK*, *tetM*, *ermA*, *ermB*, *aac(6′)–aph(2”)*, *aph(3′)-IIIa*, and *ant(9)–Ia,* compared to ST5-IV, which was isolated from healthy individuals and carried *blaZ*, *tetK, ermC*, *aph(3′)-IIIa*, and *ant(6)–Ia* [102]. Both STs did not harbor the PVL genes but carried adhesion-associated genes (fibronectin, elastin fibrinogen, biofilm production), leukocidins, and hemolysins genes. Interestingly, the *cna* gene (collagen-binding adhesin) responsible for necrotizing pneumonia was only present in ST239-III, whereas ST5-IV harbored more staphylococcal enterotoxin genes (*seg*, *sei*, *sem*, *sen*, *seo* and *seu*). Prevalence of isolated MRSAs between 2012 to 2013 was 8%, with the presence of SCC*mec* IV, ST88, ST6, and ST59 clones. These STs were more antibiotic susceptible, with the presence of virulence genes, such as *clfA*, *eno*, *fnbA*, *fnbB*, *hld*, *hlg*, *hlg2,* and *ebpS-v*. Interestingly, ST59-IV MRSA possessed the most resistance genes (*blaZ*, *ermB*, *aac(60)-Ie-aph(200)-Ia)* whereas ST6-IV only carried *blaZ*; PVL-positive ST88-IV was found to carry *blaZ* and *tetK* [105]. Collectively, these reports suggest that the epidemiology of MRSA throughout Myanmar is changing and might be dominated by CA-MRSA clones in the future.

In a follow-up study to investigate the clonal diversity and characteristics of MRSAs among pediatric patients in Yangon Children’s Hospital [106], MRSA prevalence was found to be higher (19.7%) than in 2017 (13.8%). PVL genes were detected in 35.4% of the MRSA isolates, where the majority belonged to ST772-V and ST361-V. Although ST361 is a rare MRSA clone, it has recently expanded steadily in the Arabian Gulf region, demonstrating its ability to disseminate [107]. Meanwhile, most MRSAs that lacked PVL genes belonged to the classic Asian MRSA clone, ST239-III, which was multidrug-resistant. Soe et al. also reported a higher prevalence of MRSA infections from nationwide data collected from different healthcare facilities in Myanmar during the same study period [108].

### 2.8. Philippines

MRSA is an important nosocomial pathogen in the Philippines. In 1985, only 3 (2.38%) of 126 *S. aureus* isolates from the Philippine General Hospital (PGH) showed resistance toward oxacillin [109]. MRSA was not considered an important nosocomial pathogen in PGH during the 1980s since the isolation rate was low, but it was later found to be rising to 53% from 1996 to 1998 [110]. From 1999 to 2003, Ontengo et al. collected *S. aureus* strains from three Manila hospitals and reported a five-year MRSA prevalence ranging from 5.9% to 21.7%, with a decreased susceptibility trend in the bacteria toward non–beta-lactam antibiotics (erythromycin, clindamycin, tetracycline, levofloxacin, and ciprofloxacin) between 1991 to 2001. Nonetheless, interestingly, there was a return to antibiotic susceptibility in the MRSAs isolated in 2002 to 2003 [111]. The number of MRSA cases in hospitals increased steadily throughout the following years, with several subsequent studies reporting rising MRSA prevalence [112,113]. A three-year study (2010 to 2012) in a tertiary hospital in Bacolod City recorded a total of 40.6% (38/94) MRSA isolates from the admitted patients with resistance only to chloramphenicol, tetracycline, and ciprofloxacin [112], and later in 2013, the MRSA prevalence from the clinical isolates in Makati Medical Center was 45.76% (108/236).

Early genotyping of MRSA strains collected between 1998 to 2003 revealed the predominance of ST241-III, a multidrug-resistant clone together with the presence of other MRSA clones, including ST254-IV and ST88-IV [23]. Both ST241-III and ST254-IV belong to the same clonal complex with ST239-III (CC8) while ST88-IV is widely disseminated in the African continent [7,114], Song et al. observed that major MRSA lineages circulating in the Philippines between 2004 and 2006 belonged to MRSA pandemic lineages, ST5-II (New York/ Japan clone) and ST30-IV (South West Pacific clone), both of which displayed distinct antibiotic resistance profiles and PVL gene carriage [52]. A MRSA surveillance at Makati Medical Center in 2013 showed that majority of MRSAs carried SCC*mec* type IV (96/108) and the PVL genes [23]. Nonetheless, the sequence types of circulating clones at the time of the study was not investigated. During the same year, another study was performed to investigate MRSA nasal carriage among healthcare workers in the pediatrics and surgery departments of the Philippine General Hospital. The prevalence of MRSA nasal carriage was 13%, with the circulating clones identified as ST5, ST30, ST1147, and ST97 [115]. The PVL-positive ST30-IV (South West Pacific clone) is of particular interest as this CA-MRSA clone caused an outbreak in UK neonatal ICUs, where the source was identified to be from a healthcare worker with travel history to Philippines. Notably, there were also a few other reports of travellers contracting CA-MRSA infections after returning from the Philippines [116,117]. Furthermore, ST97 has also been linked to hospital outbreaks and has been commonly found in livestock (LA-MRSA lineage) [118,119,120]. In the country’s first MRSA genomic surveillance conducted on isolates with broad geographic representation, Masim et al. discovered the predominance of clonal complex 30 (CC30) with SCC*mec* type IV and the PVL genes in MRSAs isolated from 2013–2014 [121]. A huge proportion of these isolates were resistant to penicillin (*bla*Z, *mec*A) and oxacillin (*mec*A), with additional resistance to sulfamethoxazole/trimethoprim (*dfrG*) or erythromycin (*ermC*, *msrA*). Only two isolates of ST30-IV were multidrug resistant with resistance to gentamicin (*aacA-aphD*), erythromycin (*ermC*, *msrA*), clindamycin (*ermC*), tetracycline (*tetM*, *tetK*), ciprofloxacin, levofloxacin (GyrA_S84L, GyrA_G106D, and GrlA_S80F mutations), and chloramphenicol (*cat*A1). Several international epidemic clones, such as CC30-spa-t019-SCC*mec*IV-PVL+, CC5-SCC*mec*-typeIV and ST239-spa-t030-SCC*mec*-typeIII also constituted the MRSA population in the country.

### 2.9. Indonesia

Similar to many Southeast Asian countries, there is a scarcity of data on MRSA from Indonesia, and molecular profiling has only been performed on a limited basis. Early molecular typing of representative MRSA strains between 1998 to 2003 documented the predominance of multidrug-resistant ST239 with SCC*mec* type IIIA [23]. Despite the absence of data on MRSA genotypes, multiple studies have indicated that the prevalence of MRSA varied significantly over time across Indonesia’s various geographic areas.

Lestari et al. performed the first nasal carriage screening of *S. aureus* between 2001 and 2002 in two large cities, Surabaya (Dr. Soetomo Hospital and another two primary health centers) and Semarang (Dr. Kariadi Hospital and one primary health center), where only 2/361 (0.6%) of the *S. aureus* cases harbored *mec*A [122]. Similarly, another study during the same period identified only one MRSA out of 329 strains (0.3%, SCC*mec* type V) [123]. This suggests that MRSA was still not widespread in the Indonesian community in the early 2000s.

From 2007 until 2011, Santosaningsih et al. performed a screening on *S. aureus* carriage from discharged surgical patients in three different hospitals located in Denpasar (Sanglah Hospital), Malang (Dr. Saiful Anwar Hospital), and Semarang (Dr. Kariadi Hospital) [124], where higher MRSA carriage rates were reported in Semarang (5.9%) and Malang (8%). SCC*mec* type III (94.4%) was the most prevalent type distributed among the three regions, with a significantly high proportion of ST239-III in Semarang. Compared to the absence of MRSA in Semarang (Dr. Kariadi Hospital) reported by the study conducted from 2001 to 2002 [123], this suggested that ST239-III had become endemic at the study site in less than 10 years.

Clinical *S. aureus* isolates from the same three hospitals along with Dr. M. Djamil Hospital in Padang were characterized during a similar period (2008–2009), with only 6.6% MRSA [124]. The predominance of SCC*mec* type III was observed among the clinical isolates, with a subset of genotyped isolates assigned to ST239. Later, in 2011, the first report of MRSA infection in Denpasar (Sanglah Hospital) was published, with eight patients being admitted with severe HA-MRSA infections [125], albeit without genotype information. Most of these MRSA cases showed resistance to beta-lactam antibiotics, but remained susceptible to non–beta-lactam groups. The endemicity of ST239-III continued to spread in Indonesia and was also reported subsequently in Malang (Dr. Saiful Anwar hospital) between 2012 and 2013 [126].

Another intriguing phenomenon was the continuous reporting of high-incidence PVL-positive methicillin-susceptible *S. aureus* (MSSA) in various Indonesian hospitals that were mostly resistant only to penicillin and tetracycline [124,127,128], which might have been a harbinger of PVL-positive MRSAs in the region. The emergence of CA-MRSA clones was observed through the reports of SCC*mec* type V in several locations: MRSA ST672-V in Semarang (Dr. Kariadi Hospital) [126] and ST772-V in Malang (Saiful Anwar Hospital) [127]. ST672-V exhibited resistance to fluoroquinolones, gentamicin, tetracycline, and trimethoprim-sulfamethoxazole and does not express any PVL genes unlike ST772-V. Interestingly, the same clones were reported as emerging CA-MRSA clones in Indian hospitals [129].

To determine MRSA prevalence in the community setting, Santosaningsih et al. conducted another study of *S. aureus* isolated from skin and soft tissue infections (SSTI) in Malang, Surabaya City, and Denpasar City from 2009 to 2010 [130]. Only 8/257 (3.1%) of the isolates were MRSA, where seven isolates were found in Surabaya (all were SCC*mec* type III with resistance to multiple antibiotics) and only one in Denpasar (SCC*mec* type IV with susceptibility to all tested non–beta-lactam antibiotics). MLST profiling revealed that the isolates with SCC*mec* type III were ST239, while SCC*mec* type IV isolates were ST1 (USA400), another highly virulent CA-MRSA clone [131]. Later, in 2012, a study conducted in two hospitals in another region (Jakarta) reported the presence of three CA-MRSA isolates with SCC*mec* type II, with resistance to ciprofloxacin, levofloxacin, clindamycin, Fosfomycin, and cotrimoxazole [132].

Following this, Kuntaman et al. discovered that 52 out of 643 (8.1%) patients from surgical and non-surgical wards in Surabaya (Dr Soetomo Hospital) carried MRSA prior to their hospital admission [133]. In contrast, the MRSA carriage rate was lower (0.8%) in surgical patients from Jakarta (Cipto Mangunkusumo Hospital) [134]. Given that Indonesia is a large country, the MRSA prevalence may vary across cities and much remains to be discovered about MRSAs circulating in the country.

### 2.10. Brunei and Timor-Leste

There are currently no published data about the molecular epidemiology of MRSA in Brunei. An 11-year surveillance study from 2000 to 2010 in RIPAS Hospital reported that the presence of MRSA among nosocomial infections was 6.4% [135]. Another study in 2013 investigated the prevalence of *S. aureus* and MRSA nasal carriage among young healthy adults [136]. The prevalence of *S. aureus* nasal carriage was approximately 22%, while MRSA carriage was not identified during the study period.

There are currently no published data on MRSA from Timor-Leste, which gained independence from Indonesian occupation in Mar 2002.

**Table 1:** The distribution of MRSA genotypes from 1980 to 2010 in Southeast Asian countries*.

Country	HA/CA MRSA	Year of Isolation/ST-SCC*mec* Type				MDR STs	Antibiotic-Resistant Gene Profile	Antibiotic Resistance (Phenotype)	Virulence Gene Profile	Location	References
		1980	1990	2000	2010						
Singapore	NA (1980, 1990, 2000–2010)	ST239-III	ST239-III	ST239-III	ST239-III	ST239-III	*ileS-2, aphA-3, aadD, aacA-aphD, tetK, tetM, dfrG, ermA*	CLI, ERY, TET, GEN CIP, SXT	NA	Singapore General Hospital, Tan Tock Seng Hospital, Changi General Hospital,	[25]
		ST8-I					*ermA, tetK, aacA-aphD*	CLI, ERY TET, GEN			
				ST22-IV	ST22-IV		*ermC, tetK*	CLI, ERY, TET, CIP			
				ST45-V	ST45-V			NA			
				ST78-IV				NA			
				ST5-II				NA			
	NA (1998,1999)		ST239-III			NA	NA	NA	NA	National University of Singapore	[22]
			ST614-II								
			ST363-NT								
	NA (1998–2003)		ST239-III	ST239-III		ST239	NA	PEN, CIP, CLR, CXM, GEN, AMC, LEV, SXT	NA	National University of Singapore	[23]
	CA (2001–2004)			ST80-IV		NA	NA	NA	PVL + ve	Singapore General Hospital	[30]
				ST30-IV					PVL + ve, *LukD-LukE −ve*		
				ST1-V					*LukD-LukE*, *seb,seh*, *γ2-hemolysin*		
				ST59-V					*sek*, *γ2/β-hemolysin*		
				ST524- V					*egc*, *γ2-hemolysin*		
	HA, CA (2004)			ST571-IV		ST239-III, ST22-IV, ST5-II	NA	susceptible to CIP, ERY	PVL − ve	Singapore General Hospital, Changi General Hospital, KK Women’s and Childrenʼs Hospital	[31]
				ST59-IV					PVL + ve		
				ST30-IV					PVL + ve		
				ST78-IV					PVL − ve		
				ST239-III					PVL + ve		
				ST22-IV					PVL + ve		
				ST5-II					PVL + ve		
	HA (1997–2004)		ST239-III	ST239-III		ST239-II	NA	ERY, CIP, SXT, GEN	NA	Singapore General Hospital, National University of Singapore	[24]
				ST5-II				ERY, CIP, TET, susceptible CLI, GEN			
				ST22-IV				ERY, CIP, susceptible to TET, CLI, GEN			
	HA, CA -2005			ST239-III		NA	*ermA, ermC* (ST22)	ERY, CIP, CLI, GEN, SXT TET	NA	5 Singapore public hospital	[26]
				ST22-IV			NA	CIP, ERY, CLI			
				ST30-IV			NA	NA			
				ST59-V			NA	TET			
				ST88-V			NA	NA			
	CA (2004–2005)			ST30-IV		NA	NA	susceptible to CIP, FA RIF, VAN	PVL + ve, *egc*, γ-hemolysin, *lukE*	Singapore General Hospital and local public hospitals	[32]
				ST8-V				susceptible to CIP, FA RIF, VAN	PVL + ve		
				ST45-V				ERY CLI GEN, susceptible to CIP, FA, RIF, VAN	PVL + ve		
				ST59-V				ERY, TET, susceptible to CIP, FA, RIF, VAN	PVL + ve		
				ST573-V				ERY CLI TET, susceptible to CIP, FA, RIF, VAN	PVL − ve		
				ST571-IV				susceptible to CIP, FA RIF, VAN	PVL − ve		
				ST524-V				NA	PVL + ve		
				ST188-V				SXT, TET, susceptible to CIP, FA, RIF, VAN	PVL − ve		
				ST80-IV				NA	PVL + ve		
				ST88-V				susceptible to CIP, FA, RIF, VAN	PVL + ve		
				ST78-IV				ERY, CLI, susceptible to CIP, FA, RIF, VAN	PVL − ve		
				ST7-V				ERY, CLI, susceptible to CIP, FA, RIF, VAN	PVL − ve		
	LA (2005)			ST398-V		ST398-V	NA	CIP, CLI, TET, ERY	γ-hemolysin	Pigs imported from Pulau Bulan, Indonesia, NUS slaughterhouse	[35]
				ST22-IV				ERY, CIP, CLI	NA		
	CA (outbreak) 2010				ST8-IV	NA	NA	CIP, ERY, CLOX except for one case	PVL + ve	Singapore International School	[33]
	HA, CA 2013				ST22-IV	NA	*ileS-2* (ST22, ST45, ST239, ST772)	high-level mupirocin-resistant MRSA (ST239, ST22, ST45)	NA	7 public sector hospitals	[28]
					ST45-IV/V						
					ST239-III						
					ST5-V						
					ST6-IV						
					ST30-IV						
					ST59-IV						
					ST772-IV						
					ST80-IV						
					ST88-IV						
					ST188-IV						
					ST217-IV						
					ST2718-III						
	HA, CA -2014				ST22	NA	NA	NA	NA	acute care hospitals, intermediate- and long-term care facilities	[27]
					ST45						
					ST239						
					ST188						
					ST239						
					ST622						
					ST59						
					ST30						
					ST72						
					ST105						
					ST398						
					ST573						
					ST361						
					ST1178						
Malaysia	NA (2003, 2008)			ST239-III		ST239-III, ST22-IV, ST772-V	NA	CIP, CLI, ERY, GEN, SXT, NET	NA	University Malaya Medical Centre	[38]
				ST772-V				CIP, ERY, GEN, SXT	PVL-ve		
				ST22-IV				CIP, ERY, CLI, SXT and more non-beta lactam	NA		
				ST6-IV				susceptible to all agents tested	NA		
				ST1178-IV				CIP, GEN, SXT	NA		
	HA, CA (2006–2007)			ST239-III/IIIA		NA	NA	NA	PVL − ve	Public hospital Klang Valley	[41]
				ST1-V					PVL + ve		
				ST772-V					PVL + ve		
	NA (2006–2008)			ST30-IV		NA	NA	susceptible to four or more non-b-lactam antibiotics	PVL + ve	Institute for Medical Research, KL (4 major referral hospitals)	[42]
				ST22-IV					PVL + ve		
				ST45-IV					PVL − ve		
				ST80-IV					PVL − ve		
				ST101-IV					PVL − ve		
				ST188-IV					PVL − ve		
				ST1284-IV					PVL − ve		
				ST1285-IV					PVL − ve		
				ST1286-IV					PVL − ve		
				ST1287 -IV					PVL − ve		
				ST1288-IV					PVL − ve		
	NA (2007–2008)			ST239-III/IIIA		NA	NA	NA	PVL + ve (0.7%), *sea, icaA/D, cna, fnbA*	Kuala Lumpur Hospital (HKL)	[40]
				ST1-V					*PVL*+ve*, sea, seh, icaA/D, cna, fnbA*		
				ST188-V					*PVL*+ve*, sea, icaA/D, cna, fnbA*		
				ST22-IVh					*PVL*−ve*, sec, seg, sei icaA/D, cna, fnbA*		
				ST7-V					*PVL*−ve*, sea, icaA/D, cna, fnbA*		
				ST1283-IIIA					*PVL*−ve*, sea, icaA/D, cna, fnbA*		
	NA (2008–2010)			ST239-III		NA	NA	NA	PVL − ve, *sea, ica, cna, fnbA, efb*	Hospital Sultanah Nur Zahirah (HSNZKT)	[39]
				ST772-V					PVL − ve, *sec*, *fnbA*		
				ST239-II					PVL − ve		
				ST30-IV					PVL − ve		
				ST1178-IV					PVL − ve		
				ST772-V					PVL + ve		
	NA (2009)			ST239-III		NA	NA	NA	NA	Hospital Canselor Tuanku Muhriz (HCTM)	[44]
				ST1178-IV							
	LA (2009)			ST9-V		ST9-V, ST1-V	NA	ERY, CFX, FOX, CIP, GEN, TET, SXT, CLI, TGC, Q/D	PVL − ve, *seb, see, seg, fnb*	Pig farms in Selangor	[50]
				ST1-V					PVL − ve, *seb, see, seg, fnb, cna*		
	NA (2011–2012)				ST239-III	NA	NA	NA	PVL − ve	University Malaya Medical Centre (UMMC)	[43]
					ST22-IV				PVL − ve		
					ST6-IV				PVL − ve		
					ST5-V				PVL − ve		
					ST508- NT				NA		
					ST772-V				PVL + ve		
					ST1- IV				PVL − ve		
					ST1137- IV				PVL − ve		
	NA (2013)				ST239-III	ST239-III	NA	PEN, FOX, ERY, CIP, CLI, GEN	PVL − ve	University Malaya Medical (UMMC)	[49]
					ST152-I			CMX, PEN, FOX	PVL − ve		
					ST6-IV			PEN, FOX	PVL − ve		
					ST22-IV			PEN, FOX, CIP	PVL − ve		
					ST30-IV			PEN, FOX, CIP	PVL − ve		
					ST1179-IV			PEN, FOX	PVL − ve		
					ST1-V			PEN, FOX, ERY, GEN, CLI	PVL − ve		
					ST45-V			PEN, FOX, ERY, CIP, CLI	PVL − ve		
					ST772-V			PEN, FOX, ERY, CIP, GEN	PVL − ve		
					ST951-V			PEN, FOX, ERY, CLI	PVL − ve		
					ST5-NT			PEN, FOX, GEN, ERY, CIP CLI	PVL − ve		
	NA (2016–2017)				ST22	NA	NA	NA	NA	Hospital Universiti Sains Malaysia (HUSM)	[45]
					ST239				*sasX*		
					ST4649				*sasX*		
Thailand	NA (1998–1999)		ST239-III			NA	NA	NA	NA	Mahidol University, Bangkok	[22]
	NA (1998–2003)			ST241-III		ST241-III	NA	PEN, CIP, LEV, CLR, CXM, SXT, GEN, AMC	NA	Mahidol University, Bangkok	[23]
				ST239-III		ST239-III		PEN, CIP, LEV, CLR, GEN, AMC, SXT			
	NA (2002–2003)			ST239-III		ST239-III	NA	CFZ, ERY, GEN, OFX, TET	NA	Srinagarind Hospital, Khon Kaen University	[57]
				ST5-II		ST5-II					
	HA, CA (2004–2006)			ST239-III		ST239-III	NA	high multidrug resistance rate	PVL − ve	Chulalongkorn University, Bangkok	[52]
	HA, CA (2005–2006)			ST239-III		ST239-III, ST9-IX, ST1429-III	*mecA, tetM*	CFZ, OXA, FOX, TET, ERY, OFX	PVL − ve, −ve *eta, etb, etd*, *tst*	Srinagarind Hospital, Khon Kaen University	[59]
				ST9-IX			*tetM, copB*				
				ST1429-III			NA				
	HA			ST239		NA	NA	NA	NA	Sappasithiprasong Hospital	[60]
	NA -2008			ST239		NA	*adD, ileS-2*	NA	*sea*	Sappasithiprasong Hospital	[61]
	LA (2010–2011)			ST764-II		ST764-II, ST9-IX		OXA, FOX, GEN, ERY, TET, CFZ, OFX	PVL − ve*, hla*	Pig farms	[71]
				ST9-IX				OXA, FOX, GEN, TET, OFX, CFZ			
	NA (2015–2016)				ST239-III	ST239-III	*ermA, ermB, ermC*	PEN, OXA, FOX, ERY, CLI, GEN, CIP	NA	Chiangrai Prachanukroh Hospital	[64]
	CA (2015–2016)				ST2885-IV	NA	*blaZ/mecA, Inu(A), tet(K), dfrG*	susceptible to TET, CLI, SXT but not β-lactam	*eta*, *hlgA, hlgB, hlgC*, *lukD, lukE*	Hua Hin Hospital	[65]
	HA (2012–2015)				ST764-II	NA	NA	CPR, CLI	PVL − ve, *lukE-lukD*, *hla, hld, hlgA*, *seg, sei, sem, sen, seo, set6 to set16*, *icaA*, *cna*, *fnbA, fnbB, ebpS*, *clfA, clfB, sdrC, sdrD, sdrE*	Thammasat University Hospital	[62]
					ST22-III				NA		
					ST239-III				NA		
	LA (2015–2017)				ST398-V	ST398-V, ST9-IX, ST4576-IX	*mecA, blaZ, tet(K), tet(M), ant(4′)-Ia, ant(6)-Ia, dfrG, erm(C), lnu(B), lsa(E), spw*	PEN, FOX, TET, STR, TMP, ERY, CLI, TIA, CIP, Q/D	NA	Pig farms central Thailand	[68]
					ST9-IX		*mecA, blaZ, ant (4)-Ia, tet(*L*), tet*(M), *aac(6″)-Ie aph(2″)-Ia, dfrG, erm(B), vga*(A)*, cfr, fexA, str*	PEN, FOX, TET, GEN, KAN, STR, TMP, CLI, TIA, CHL, CIP, ERY, SXT, LZD, Q/D			
					ST4576-IX		*mecA, blaZ, tet, aac(6)-Ie aph(2″)- (M)- Ia, dfrG, vga, cat, str*	PEN, FOX, TET, GEN, KAN, STR, TMP, CLI, TIA, Q/D, CHL, CIP			
	LA (2017–2018)				ST9-IX/NT	ST9-IX/NT, ST398-V, ST779-IV, ST5639-IX	NA	AMP, OXA, FOX, CHL, CLI, ERY, CIP, ENR, GEN, TET, SXT	NA	Pig slaughterhouse, market	[72]
					ST398-V			AMP, OXA, FOX, CLI, ERY, CIP, ERY, TET			
					ST779-IV			AMP, OXA, FOX, CHL, CLI, CIP, ERY, GEN, TET			
					ST5639-IX			AMP, OXA, FOX, CHL, TET			
	HA (2019)				ST9-IX		*mecA, blaZ, aac(69)-aph (20), aadD, ant(6)-Ia, lsa(E), dfrG, tet(M), fexA, lnu(B*	NA	*aur, seg, sei, sem, sen, seo, seu, hlgA, hlgB, hlgC*	Northern Thailand	[70]
Vietnam	HA (1998–1999)		ST239-III			NA	NA	NA	NA	University of Medicine and Pharmacy, Ho Chi Minh	[22]
			ST241-III								
			ST346-II								
	NA (1998–2003)		ST239-III	ST239-III		ST239-III	NA	PEN, CIP, LEV, CLR, CXM, GEN, AMC	NA	Nhi Dong 2 Children’s Hospital, Ho Chi-Minh	[23]
			ST241-III	ST241-III		ST241-III		PEN, CIP, LEV, CLR, CXM, SXT, GEN, AMC			
	HA, CA (2004–2006)			ST239-III		ST239-III	NA	high multidrug resistance rate	PVL − ve	University of Medicine and Pharmacy, Ho Chi Minh	[52]
				ST59-IV		ST59-IV		high resistance rates to non–beta-lactam antibiotics	NA		
	CA (outbreak) -2006			ST59-IV/V		NA	*blaZ*	susceptible to most other agents tested	PVL + ve, *hla, hlagACB, hld*, *seg, seb*, *clfAB, sdrDE*, *cap8*	Pediatric Hospital, Ho Chi Minh	[77]
				ST1-IV			*blaZ*		PVL + ve, *hla, hlagACB, hld*, *seg,sea, sak, sek, seccna*, *clfAB, sdrDE*, *cap8*		
	HA, CA (2009–2012)			ST59	ST59	NA	NA	NA	PVL + ve (ST59, ST88, ST121) PVL − ve (ST239, ST188, ST8, ST45, ST25)	Dong Da and Ba Vi districts (northern Vietnam) National Hospital of Tropical Diseases (NHTD), Hanoi.	[83]
				ST188	ST188						
				ST45	ST45						
				ST8	ST239						
				ST834	ST88						
				ST25	ST121						
	HA, CA (2011–2012)				ST239-II/III	ST5-II, ST239-II/III, ST45-IV, ST59-IV, ST88-IV, ST2808-IV	*mecA, aacA/ aphD, ermA/B/C, tetK/M*	CIP, ERY, GEN, TET	NA	National Hospital of Pediatric, Hanoi Children’s Hospital No. 2, Ho Chi Minh City	[81]
					ST5-II						
					ST319-IV						
					ST45-IV						
					ST2808-IV						
					ST59-III						
					ST88-IV						
	HA (2013–2014)				ST188-III/V	ST188-III/IV, ST239-II	*mecA, tetK/M, aacA/aphD, ermA/B/C*	CIP, ERY, GEN, TET	NA	Bach Mai General Hospital 103 Military Hospital Hue General Hospital 175 General Hospital	[75]
					ST239-II						
	
	
Cambodia	CA (sporadic) (2006–2007)			ST834-IV				CIP, SXT, RIF	PVL − ve	Angkor Hospital for Children, Siem Reap	[91]
				ST121-V		NA	NA	susceptible to CIP, SXT, RIF	PVL + ve		
	CA -2008			ST834		NA	NA	ERY, TET, SXT, RIF	NA	Angkor Hospital for Children, Siem Reap	[95]
				ST121							
				ST188							
				ST45							
				ST9							
Laos	HA, CA (2012–2014)				ST239-III	ST239-III	*blaZ, ermA, tetK, tetM, aacA-aphD*	PEN, ERY, TET, GEN, SXT, CIP	PVL − ve, *cap8*, *sea, sek/q*, agrI, *scn+ sak*	Mahosot Hospital, Vientiane	[101]
					ST2885-V		*blaZ, tetK*	PEN, TET	PVL − ve, *cap5, etA*, agrIV, *scn+ sak*		
					ST59/952-V		*blaZ, ermB, tetK, cat*	PEN, ERY, TET, CHL	PVL + ve, *cap8*, *seb, sek/q*, agrI, *scn+ chp*		
					ST2250-IV		*blaZ*	PEN, TET	PVL − ve, *scn+ sak*		
					CC398-V		*blaZ, ermA, tetK*	PEN, ERY, TET	PVL + ve, *cap5*, *scn+ chp+ sak*		
Myanmar	HA, CA			ST239-III		ST239-III	*blaZ, tetK, tetM, ermA, ermB, aac(6′)–aph (2″), aph(3′)-IIIa, ant(9)–Ia*	OXA, FOX, GEN, ERY, TET, CIP	PVL − ve, *lukE-lukD*, hemolysins *hlah, hlb, hlg, hlg–v, hld*, *sea, selk, selq*, *set8/9, set10, set13*, *icaA, icaD*, *can, eno*, *fnbA, fnbB, ebpS, clfA, clfB, fib, sdrC, sdrD, sdrE*	general hospital in Yangon	[102]
	(2007–2008)			ST5-IV		ST5-IV	*blaZ, tetK, ermC, aph (3′)-IIIa, ant(6)–Ia*	OXA, AMP, FOX, TET, ERY	PVL − ve, *lukE-lukD*, *hlah, hlb, hlg, hlg–v, hld*, *seg, sei, selm, seln, selo, seluicaA, icaD*, *eno*, *fnbA, fnbB, ebpS*, *clfA, clfB, fib, sdrC, sdrD*	hotel workers in Yangon	
	HA (2012–2013)				ST88-IV	NA	*blaZ, tetK*	OXA, AMP, GEN, ERY	PVL + ve, l*ukE-lukD, hld, hlg, hlg2*, *sei, sek, seq*, *fnbA, fnbB, ebpS*, *clfA,clfB, fib, sdrC, sdrD, sdrE*, *eno*	North Okkalapa General Hospital, Yangon	[105]
					ST6-IV		*blaZ*		PVL − ve, *lukE-lukD*, *hld, hlg, hla, hlg2*, *sea*, *fnbA, fnbB, ebpS*, *clfA, fib, sdrC, sdrD, sdrE*, *eno*, *cna*		
					ST59-IV		*blaZ, ermB, aac(6)-Ie-aph(2)-Ia*		PVL − ve, *hld, hlg, hla, hlg2, seb, sek, seq*, *fnbA, fnbB, ebpS*, *clfA, fib, sdrC, sdrD, sdrE*, *eno*		
	HA, CA (2017–2019)				ST772-V	ST772-IV, ST8-IV	NA	OXA, FOX, AMP, CFZ, GEN, ERY, CLI, SXT, LEV	PVL + ve, *seg, sei, sem, sen, seo*	Pinlon Hospital, Yangon	[103]
					ST4599-V			OXA, FOX, AMP, GEN, SXT, LEV	PVL + ve, *seg, sei, sem, sen, seo*		
					ST88-IV			OXA, FOX, AMP	PVL + ve		
					ST8-IV			OXA, FOX, AMP, CFZ, ERY, CLI, LEV	PVL + ve		
					ST22-IV/V			OXA, FOX, AMP, CLI, ERY, GEN, LEV, SXT	PVL − ve, *seg, sei, sem, sen, seo*		
	NA				ST239-III	ST239-III	NA	high multidrug resistance rate	PVL − ve	Yangon Children’s Hospital	[106]
					ST361-V				PVL + ve		
					ST772-V				PVL + ve		
Philippines	NA		ST241-III			NA	NA	NA	NA	Research Institute for Tropical Medicine, Manila	[22]
	(1998–1999)		ST254-II								
	NA		ST241-III	ST241-III		ST241-III	NA	PEN, CIP, CFX, SXT, GEN, AMC, LEV, CLA	NA	Research Institute for Tropical Medicine, Manila	[23]
	(1998–2003)		ST88-IV	ST88-IV				PEN, GEN, AMC			
	HA, CA			ST30-IV			NA	Susceptible to non–beta-lactam antibiotics	PVL + ve	Research Institute for Tropical Medicine, Manila	[52]
	(2004–2006)			ST5-II		ST5-II		high multidrug resistance rate	PVL − ve		
	HA (2013)				ST30-I/IV	NA	NA	NA	PVL + ve	Philippine General Hospital	[115]
					ST97-IV				PVL + ve		
					ST1147-I				PVL − ve		
					ST5-IV				PVL − ve		
	HA, CA (2013–2014)				ST30-IV	NA	*bla*Z*, mec*A*, aacA_aphD, ermC, tetM, tetK,* Gyr*A_S84L,* GyrA_G106D*,* GrlA_S80F*, catA1, sdr*M*, ileS_2, sdrM, dfrG, msr*A	PEN, OXA with resistance to SXT, ERY	PVL + ve	Collection by DOH-ARSP	[121]
					ST5-IV	NA	NA	NA	PVL − ve (ST239)		
					ST239-III						
					ST1-IV						
					ST121						
					ST1456-IV						
					ST1457-IV						
					ST1649-IV						
					ST834-IV						
					ST97-IV						
					ST5-IV						
					ST30-IV						
					ST508-IV						
					ST88						
Indonesia	NA (1998–1999)		ST239-III			NA	NA	NA	NA	University of Indonesia, Jakarta	[22]
	NA (1998–2003)		ST239-III	ST239-III		ST239-III	NA	PEN, CIP, CFX, SXT, GEN, AMC, LEV	NA	University of Indonesia, Jakarta	[23]
	NA (2007–2011)			ST239-III	ST239-III	NA	NA	NA	PVL − ve	Sanglah Hospital, Dr. Kariadi Hospital, Dr. Saiful Anwar Hospital	[124]
	NA (2008–2009)			ST239-III		ST239-III ST672-V	NA	PEN, CIP, LEV, GEN, TOB, TET	PVL − ve	Sanglah Hospital, Dr. Kariadi Hospital, Dr. Saiful Anwar Hospital, Dr. M. Djamil Hospital	[127]
				ST672-V				PEN, CIP, LEV, GEN, TET, SXT			
	HA, CA (2009–2010)			ST239-III		ST239-III	NA	CIP, LEV, TET, SXT, GEN, ERY	PVL − ve, *eta etb*-ve	Malang, Surabaya city, Denpasar city	[130]
				ST1-IV				Susceptible to all non–beta-lactam antibiotics			
	HA, CA (2012–2013)				ST239-III	NA	NA	NA	PVL*+*ve (ST722-V)	Dr. Saiful Anwar Hospital	[126]
					ST772-V						

* NT: Non-typeable; NA: Not available in the literature; AMC, amoxicillin/clavulanic acid; AMP, ampicillin; CHL, chloramphenicol; CFX, ceftriaxone; CFZ, cefazolin; CPR, cefpirome; CIP, ciprofloxacin; CLI, clindamycin; CLR, clarithromycin; CXM, cefuroxime; CLOX, cloxacillin; CXM, cefuroxime; ERY, erythromycin; FA, fusidic acid; FOX, cefoxitin; GEN, gentamicin; Q/D, quinupristin/dalfopristine; TGC, tigecycline; LEV, levofloxacin; STR, streptomycin; SXT, trimethoprim/sulfamethoxazole; KAN, kanamycin; TET, tetracycline; TOB, tobramycin; PEN, penicillin; TIA, tiamulin; TMP, trimethoprim; NET, netilmicin; OFX, ofloxacin; LZD, linezolid.

## 3. Conclusions

This review highlights the molecular epidemiology and distribution of MRSA in Southeast Asia. MRSA epidemiology was found to have shifted over the years from the 1970s until recent years, and widespread distribution of various MRSA clones can be observed throughout the region. It remains difficult to uncover the true prevalence and predominant MRSA genotypes in the region due to differences in laboratory and surveillance capacities of each country. Notably, while antibiotic selection pressure has been reported to hypothetically bring about the pandemic spread of ST239, fitter clones, such as the ST22, have come to dominate in Southeast Asia, even though it exhibited less resistance to non–beta-lactam and non-fluoroquinolone antibiotics. Nonetheless, studies on LA-MRSA remains significantly few. Given that clones of the pathogen have successfully achieved pandemic spread, it is probable that MRSAs in the region will continue to evolve and disseminate, mirroring the situation on other continents. Prudent antibiotic stewardship by clinicians, pharmacists, and other healthcare professionals, together with periodical genome surveillance of MRSA in both humans and livestock by epidemiologists will be crucial to monitor, understand, and predict the pathogen’s dissemination and transmission into the future.

## Data Availability

Not applicable.

## References

[1] Liverani M., Oliveira Hashiguchi L., Khan M., Coker R., Jamrozik E., Selgelid M. (2020). Antimicrobial Resistance and the Private Sector in Southeast Asia. Ethics and Drug Resistance: Collective Responsibility for Global Public Health.

[2] Nhung N.T., Cuong N.V., Thwaites G., Carrique-Mas J. (2016). Antimicrobial Usage and Antimicrobial Resistance in Animal Production in Southeast Asia: A Review. Antibiotics.

[3] Murray C.J., Ikuta K.S., Sharara F., Swetschinski L., Robles Aguilar G., Gray A., Han C., Bisignano C., Rao P., Wool E. (2022). Global Burden of Bacterial Antimicrobial Resistance in 2019: A Systematic Analysis. Lancet.

[4] Otto M. (2012). Molecular Insight into How MRSA Is Becoming Increasingly Dangerous. Virulence.

[5] Lakhundi S., Zhang K. (2018). Methicillin-Resistant Staphylococcus Aureus: Molecular Characterization, Evolution, and Epidemiology. Clin. Microbiol. Rev..

[6] Enright M.C., Day N.P., Davies C.E., Peacock S.J., Spratt B.G. (2000). Multilocus Sequence Typing for Characterization of Methicillin-Resistant and Methicillin-Susceptible Clones of Staphylococcus Aureus. J. Clin. Microbiol..

[7] Enright M.C., Robinson D.A., Randle G., Feil E.J., Grundmann H., Spratt B.G. (2002). The Evolutionary History of Methicillin-Resistant Staphylococcus Aureus (MRSA). Proc. Natl. Acad. Sci. USA.

[8] Lindsay J.A. (2013). Hospital-Associated MRSA and Antibiotic Resistance—What Have We Learned from Genomics?. Int. J. Med. Microbiol..

[9] Rae N., Jarchow-MacDonald A., Nathwani D., Marwick C.A. (2016). MRSA: Treating People with Infection. BMJ Clin. Evid..

[10] Sabtu N., Enoch D.A., Brown N.M. (2015). Antibiotic Resistance: What, Why, Where, When and How?. Br. Med. Bull..

[11] Kale P., Dhawan B. (2016). The Changing Face of Community-Acquired Methicillin-Resistant Staphylococcus Aureus. Indian J. Med. Microbiol..

[12] O’Brien F.G., Lim T.T., Chong F.N., Coombs G.W., Enright M.C., Robinson D.A., Monk A., Saïd-Salim B., Kreiswirth B.N., Grubb W.B. (2004). Diversity among Community Isolates of Methicillin-Resistant Staphylococcus Aureus in Australia. J. Clin. Microbiol..

[13] Wijaya L., Hsu L.Y., Kurup A. (2006). Community-Associated Methicillin-Resistant Staphylococcus Aureus: Overview and Local Situation. Ann. Acad. Med. Singap..

[14] Chen C., Wu F. (2020). Livestock-Associated Methicillin-Resistant Staphylococcus Aureus (LA-MRSA) Colonisation and Infection among Livestock Workers and Veterinarians: A Systematic Review and Meta-Analysis. Occup. Environ. Med..

[15] Van Loo I., Huijsdens X., Tiemersma E., De Neeling A., Van De Sande-Bruinsma N., Beaujean D., Voss A., Kluytmans J. (2007). Emergence of Methicillin-Resistant Staphylococcus Aureus of Animal Origin in Humans. Emerg. Infect. Dis..

[16] Chen C.J., Huang Y.C. (2014). New Epidemiology of Staphylococcus Aureus Infection in Asia. Clin. Microbiol. Infect..

[17] Coker R.J., Hunter B.M., Rudge J.W., Liverani M., Hanvoravongchai P. (2011). Emerging Infectious Diseases in Southeast Asia: Regional Challenges to Control. Lancet.

[18] Sartelli M., Hardcastle T.C., Catena F., Chichom-Mefire A., Coccolini F., Dhingra S., Haque M., Hodonou A., Iskandar K., Labricciosa F.M. (2020). Antibiotic Use in Low and Middle-Income Countries and the Challenges of Antimicrobial Resistance in Surgery. Antibiotics.

[19] Iskandar K., Molinier L., Hallit S., Sartelli M., Hardcastle T.C., Haque M., Lugova H., Dhingra S., Sharma P., Islam S. (2021). Surveillance of Antimicrobial Resistance in Low- and Middle-Income Countries: A Scattered Picture. Antimicrob. Resist. Infect. Control.

[20] Lim W.W., Wu P., Bond H.S., Wong J.Y., Ni K., Hong W., Jit M., Cowling B.J. (2019). Determinants of MRSA Prevalence in the Asia Pacific Region: A Systematic Review and Meta-Analysis. J. Glob. Antimicrob. Resist..

[21] Hsu L.Y., Tan T.Y., Jureen R., Koh T.H., Krishnan P., Lin R.T.P., Tee N.W.S., Tambyah P.A. (2007). Antimicrobial Drug Resistance in Singapore Hospitals. Emerg. Infect. Dis..

[22] Chongtrakool P., Ito T., Ma X.X., Kondo Y., Trakulsomboon S., Tiensasitorn C., Jamklang M., Chavalit T., Song J.H., Hiramatsu K. (2006). Staphylococcal Cassette Chromosome Mec (SCCmec) Typing of Methicillin-Resistant Staphylococcus Aureus Strains Isolated in 11 Asian Countries: A Proposal for a New Nomenclature for SCCmec Elements. Antimicrob. Agents Chemother..

[23] Kwan S.K., Lee J.Y., Ji Y.S., Won S.O., Kyong R.P., Nam Y.L., Song J.H. (2005). Distribution of Major Genotypes among Methicillin-Resistant Staphylococcus Aureus Clones in Asian Countries. J. Clin. Microbiol..

[24] Hsu L.Y., Koh T.H., Singh K., Kang M.L., Kurup A., Tan B.H. (2005). Dissemination of Multisusceptible Methicillin-Resistant Staphylococcus Aureus in Singapore. J. Clin. Microbiol..

[25] Hsu L.Y., Harris S.R., Chlebowicz M.A., Lindsay J.A., Koh T.H., Krishnan P., Tan T.Y., Hon P.Y., Grubb W.B., Bentley S.D. (2015). Evolutionary Dynamics of Methicillin-Resistant Staphylococcus Aureus within a Healthcare System. Genome Biol..

[26] Hsu L.Y., Loomba-Chlebicka N., Koh Y.L., Tan T.Y., Krishnan P., Lin R.T.P., Tee N.W.S., Fisher D.A., Koh T.H. (2007). Evolving EMRSA-15 Epidemic in Singapore Hospitals. J. Med. Microbiol..

[27] Chow A., Lim V.W., Khan A., Pettigrew K., Lye D.C.B., Kanagasabai K., Phua K., Krishnan P., Ang B., Marimuthu K. (2017). MRSA Transmission Dynamics among Interconnected Acute, Intermediate-Term, and Long-Term Healthcare Facilities in Singapore. Clin. Infect. Dis..

[28] Hon P.Y., Koh T.H., Tan T.Y., Krishnan P., Leong J.W.Y., Jureen R., Chan J., Tee N.W.S., Murugesh J., Chan K.S. (2014). Changing Molecular Epidemiology and High Rates of Mupirocin Resistance among Meticillin-Resistant Staphylococcus Aureus in Singaporean Hospitals. J. Glob. Antimicrob. Resist..

[29] Effelsberg N., Stegger M., Peitzmann L., Altinok O., Coombs G.W., Pichon B., Kearns A., Randad P.R., Heaney C.D., Bletz S. (2020). Global Epidemiology and Evolutionary History of Staphylococcus Aureus ST45. J. Clin. Microbiol..

[30] Hsu L.Y., Tristan A., Koh T.H., Bes M., Etienne J., Kurup A., Tan T.T., Tan B.H. (2005). Community-Associated Methicillin-Resistant Staphylococcus Aureus, Singapore. Emerg. Infect. Dis..

[31] Hsu L.Y., Koh T.H., Tan T.Y., Ito T., Ma X.X., Lin R.T., Tan B.H. (2006). Emergence of Community-Associated Aureus in Singapore: A Further Six Cases. Singap. Med. J..

[32] Hsu L.Y., Koh Y.L., Chlebicka N.L., Tan T.Y., Krishnan P., Lin R.T.P., Tee N., Barkham T., Koh T.H. (2006). Establishment of ST30 as the Predominant Clonal Type among Community-Associated Methicillin-Resistant Staphylococcus Aureus Isolates in Singapore. J. Clin. Microbiol..

[33] Grant D., Koh T.H.S., Tan Y.E., Hsu L.Y., Kurup A., Donahue S.K., Mann J., Fisher D. (2013). An Outbreak of Community Associated Methicillin Resistant Staphylococcus Aureus Subtype USA300 at an International School in Singapore. Ann. Acad. Med. Singap..

[34] Stegger M., Wirth T., Andersen P.S., Skov R.L., De Grassi A., Simões P.M., Tristan A., Petersen A., Aziz M., Kiil K. (2014). Origin and Evolution of European Community-Acquired Methicillin- Resistant Staphylococcus Aureus. mBio.

[35] Sergio D.M.B., Koh T.H., Hsu L.-Y., Ogden B.E., Goh A.L.H., Chow P.K.H. (2007). Investigation of Meticillin-Resistant Staphylococcus Aureus in Pigs Used for Research. J. Med. Microbiol..

[36] Cheong I., Tan S.C., Wong Y.H., Zainudin B.M.Z., Rahman M.Z.A. (1994). Methicillin-Resistant Staphylococcus Aureus (MRSA) in a Malaysian Hospital. Med. J. Malays..

[37] Lim V.K.E., Zulkifli H.I. (1987). Methicillin Resistant Staphylococcus Aureus in a Malaysian Neonatal Unit. Singap. Med. J..

[38] Lim K.T., Hanifah Y.A., Mohd Yusof M.Y., Ito T., Thong K.L. (2013). Comparison of Methicillin-Resistant Staphylococcus Aureus Strains Isolated in 2003 and 2008 with an Emergence of Multidrug Resistant ST22: SCCmec IV Clone in a Tertiary Hospital, Malaysia. J. Microbiol. Immunol. Infect..

[39] Lim K.T., Yeo C.C., Suhaili Z., Thong K.L. (2012). Comparison of Methicillin-Resistant and Methicillin-Sensitive Staphylococcus Aureus Strains Isolated from a Tertiary Hospital in Terengganu, Malaysia. Jpn. J. Infect. Dis..

[40] Ghaznavi-Rad E., Shamsudin M.N., Sekawi Z., Khoon L.Y., Aziz M.N., Hamat R.A., Othman N., Chong P.P., Van Belkum A., Ghasemzadeh-Moghaddam H. (2010). Predominance and Emergence of Clones of Hospital-Acquired Methicillin-Resistant Staphylococcus Aureus in Malaysia. J. Clin. Microbiol..

[41] Neela V., Ghasemzadeh Moghaddam H., Van Belkum A., Horst-Kreft D., Mariana N.S., Ghaznavi Rad E. (2010). First Report on Methicillin-Resistant Staphylococcus Aureus of Spa Type T037, Sequence Type 239, SCCmec Type III/IIIA in Malaysia. Eur. J. Clin. Microbiol. Infect. Dis..

[42] Ahmad N., Ruzan I.N., Ghani M.K.A., Hussin A., Nawi S., Aziz M.N., Maning N., Eow V.L.K. (2009). Characteristics of Community- and Hospital-Acquired Meticillin-Resistant Staphylococcus Aureus Strains Carrying SCCmec Type IV Isolated in Malaysia. J. Med. Microbiol..

[43] Sit P.S., Teh C.S.J., Idris N., Sam I.C., Syed Omar S.F., Sulaiman H., Thong K.L., Kamarulzaman A., Ponnampalavanar S. (2017). Prevalence of Methicillin-Resistant Staphylococcus Aureus (MRSA) Infection and the Molecular Characteristics of MRSA Bacteraemia over a Two-Year Period in a Tertiary Teaching Hospital in Malaysia. BMC Infect. Dis..

[44] Noordin A., Sapri H.F., Sani N.A.M., Leong S.K., Tan X.E., Tan T.L., Zin N.M., Neoh H.M., Hussin S. (2016). Antimicrobial Resistance Profiling and Molecular Typing of Methicillin-Resistant Staphylococcus Aureus Isolated from a Malaysian Teaching Hospital. J. Med. Microbiol..

[45] Nurhafiza N., Siti Asma H., Azian H., Foo P., Yasmin K., Chan Y. (2022). Distribution of SasX, QacA/B and MupA Genes and Determination of Genetic Relatedness of Methicillin-Resistant Staphylococcus Aureus among Clinical Isolates and Nasal Swab Samples from the Same Patients in a Hospital in Malaysia. Singap. Med. J..

[46] Ismail M.A.H., Kamarudin N., Samat M.N.A., Rahman R.M.F.R.A., Saimun S., Tan T.L., Neoh H.M. (2021). Methicillin-Resistant Staphylococcus Aureus (Mrsa) Clonal Replacement in a Malaysian Teaching Hospital: Findings from an Eight-Year Interval Molecular Surveillance. Antibiotics.

[47] Pardos De La Gandara M., Curry M., Berger J., Burstein D., Della-Latta P., Kopetz V., Quale J., Spitzer E., Tan R., Urban C. (2016). MRSA Causing Infections in Hospitals in Greater Metropolitan New York: Major Shift in the Dominant Clonal Type between 1996 and 2014. PLoS ONE.

[48] Albrecht N., Jatzwauk L., Slickers P., Ehricht R., Monecke S. (2011). Clonal Replacement of Epidemic Methicillin-Resistant Staphylococcus Aureus Strains in a German University Hospital over a Period of Eleven Years. PLoS ONE.

[49] Sit P.S., Teh C.S.J., Idris N., Ponnampalavanar S. (2018). Methicillin-Resistant Staphylococcus Aureus (MRSA) Bacteremia: Correlations between Clinical, Phenotypic, Genotypic Characteristics and Mortality in a Tertiary Teaching Hospital in Malaysia. Infect. Genet. Evol..

[50] Neela V., Mohd Zafrul A., Mariana N.S., van Belkum A., Liew Y.K., Rad E.G. (2009). Prevalence of ST9 Methicillin-Resistant Staphylococcus Aureus among Pigs and Pig Handlers in Malaysia. J. Clin. Microbiol..

[51] Neela V., Ghaznavi Rad E., Ghasemzadeh Moghaddam H., nor Shamsudin M., van Belkum A., Karunanidhi A. (2013). Frequency of Methicillin Resistant Staphylococcus Aureus in the Noses of Malaysian Chicken Farmers and Their Chicken. Iran. J. Vet. Res..

[52] Aklilu E., Hui Ying C. (2020). First MecC and MecA Positive Livestock-Associated Methicillin Resistant Staphylococcus Aureus (MecC MRSA/LA-MRSA) from Dairy Cattle in Malaysia. Microorganisms.

[53] Danchaivijitr S., Trakulsomboon S. (1989). Patients Colonized by Antibiotic Resistant Bacteria. A Potential Source of Infections in the Medical Wards. J. Med. Assoc. Thail..

[54] Choojitr W., Ruangkris T. (1995). Surgical Wound Infections in Gynaecology at Rajvithi Hospital 1989-1990. J. Med. Assoc. Thail. Chotmaihet Thangphaet.

[55] Danchivijitr S., Chantrasakul C., Chokloikaew S., Trakoolsomboon S. (1995). An Outbreak of Methicillin-Resistant Staphylococcus Aureus (M.R.S.A.) in a Burn Unit. J. Med. Assoc. Thail..

[56] Song J.H., Hsueh P.R., Chung D.R., Ko K.S., Kang C.I., Peck K.R., Yeom J.S., Kim S.W., Chang H.H., Kim Y.S. (2011). Spread of Methicillin-Resistant Staphylococcus Aureus between the Community and the Hospitals in Asian Countries: An ANSORP Study. J. Antimicrob. Chemother..

[57] Lulitanond A., Chanawong A., Sribenjalux P., Wilailuckana C., Kaewkes W., Vorachit M., Ito T., Hiramatsu K. (2010). Preliminary Report of SCCmec-Types and Antimicrobial Susceptibilities of Methicillin-Resistant Staphylococcus Aureus Isolates from a University Hospital in Thailand. Southeast Asian J. Trop. Med. Public Health.

[58] Monecke S., Coombs G., Shore A.C., Coleman D.C., Akpaka P., Borg M., Chow H., Ip M., Jatzwauk L., Jonas D. (2011). A Field Guide to Pandemic, Epidemic and Sporadic Clones of Methicillin-Resistant Staphylococcus Aureus. PLoS ONE.

[59] Lulitanond A., Ito T., Li S., Han X., Ma X.X., Engchanil C., Chanawong A., Wilailuckana C., Jiwakanon N., Hiramatsu K. (2013). ST9 MRSA Strains Carrying a Variant of Type IX SCCmec Identified in the Thai Community. BMC Infect. Dis..

[60] Feil E.J., Nickerson E.K., Chantratita N., Wuthiekanun V., Srisomang P., Cousins R., Pan W.B., Zhang G., Xu B.L., Day N.P.J. (2008). Rapid Detection of the Pandemic Methicillin-Resistant Staphylococcus Aureus Clone ST 239, a Dominant Strain in Asian Hospitals. J. Clin. Microbiol..

[61] Tong S.Y.C., Holden M.T.G., Nickerson E.K., Cooper B.S., Koser C.U., Cori A., Jombart T., Cauchemez S., Fraser C., Wuthiekanun V. (2015). Genome Sequencing Defines Phylogeny and Spread of Methicillin-Resistant Staphylococcus Aureus in a High Transmission Setting. Genome Res..

[62] Kondo S., Phokhaphan P., Tongsima S., Ngamphiw C., Phornsiricharoenphant W., Ruangchai W., Disratthakit A., Tingpej P., Mahasirimongkol S., Lulitanond A. (2022). Molecular Characterization of Methicillin-Resistant Staphylococcus Aureus Genotype ST764-SCCmec Type II in Thailand. Sci. Rep..

[63] Takano T., Hung W.-C., Shibuya M., Higuchi W., Iwao Y., Nishiyama A., Reva I., Khokhlova O.E., Yabe S., Ozaki K. (2013). A New Local Variant (ST764) of the Globally Disseminated ST5 Lineage of Hospital-Associated Methicillin-Resistant Staphylococcus Aureus (MRSA) Carrying the Virulence Determinants of Community-Associated MRSA. Antimicrob Agents Chemother.

[64] Kitti T., Seng R., Saiprom N., Thummeepak R., Chantratita N., Boonlao C., Sitthisak S. (2018). Molecular Characteristics of Methicillin-Resistant Staphylococci Clinical Isolates from a Tertiary Hospital in Northern Thailand. Can. J. Infect. Dis. Med. Microbiol..

[65] Santimaleeworagun W., Preechachuawong P., Samret W., Jitwasinkul T. (2021). The First Report of a Methicillin-Resistant Staphylococcus Aureus Isolate Harboring Type IV SCCmec in Thailand. Pathogens.

[66] Mekviwattanawong S., Srifuengfung S., Chokepaibulkit K., Lohsiriwat D., Thamlikitkul V. (2006). Epidemiology of Staphylococcus Aureus Infections and the Prevalence of Infection Caused by Community-Acquired Methicillin-Resistant Staphylococcus Aureus in Hospitalized Patients at Siriraj Hospital. J. Med. Assoc. Thai..

[67] Li S., Skov R.L., Han X., Larsen A.R., Larsen J., Sørum M., Wulf M., Voss A., Hiramatsu K., Ito T. (2011). Novel Types of Staphylococcal Cassette Chromosome Mec Elements Identified in Clonal Complex 398 Methicillin-Resistant Staphylococcus Aureus Strains. Antimicrob. Agents Chemother..

[68] Chanchaithong P., Perreten V., Am-In N., Lugsomya K., Tummaruk P., Prapasarakul N. (2019). Molecular Characterization and Antimicrobial Resistance of Livestock-Associated Methicillin-Resistant Staphylococcus Aureus Isolates from Pigs and Swine Workers in Central Thailand. Microb. Drug Resist..

[69] Chuang Y.Y., Huang Y.C. (2015). Livestock-Associated Meticillin-Resistant Staphylococcus Aureus in Asia: An Emerging Issue?. Int. J. Antimicrob. Agents.

[70] Chopjitt P., Wongsurawat T., Jenjaroenpun P., Boueroy P., Kamjumphol W., Hatrongjit R., Kerdsin A. (2021). Draft Genome Sequence of Methicillin-Resistant Staphylococcus Aureus Harboring Staphylococcal Cassette Chromosome Mec Type IX, Isolated from a Fatal Bacteremic Pneumonia Case. Microbiol. Resour. Announc..

[71] Sinlapasorn S., Lulitanond A., Angkititrakul S., Chanawong A., Wilailuckana C., Tavichakorntrakoo R., Chindawong K., Seelaget C., Krasaesom M., Chartchai S. (2015). SCCmec IX in Meticillin-Resistant Staphylococcus Aureus and Meticillin-Resistant Coagulase-Negative Staphylococci from Pigs and Workers at Pig Farms in Khon Kaen, Thailand. J. Med. Microbiol..

[72] Tanomsridachchai W., Changkaew K., Changkwanyeun R., Prapasawat W., Intarapuk A., Fukushima Y., Yamasamit N., Kapalamula T.F., Nakajima C., Suthienkul O. (2021). Antimicrobial Resistance and Molecular Characterization of Methicillin-Resistant Staphylococcus Aureus Isolated from Slaughtered Pigs and Pork in the Central Region of Thailand. Antibiotics.

[73] Phokhaphan P., Tingpej P., Apisarnthanarak A., Kondo S. (2017). Prevalence and Antibiotic Susceptiblity of Methicillin Resistant Staphylococcus Aureus, Collected at Thammasat University Hospital, Thailand, August 2012–July 2015. Southeast Asian J. Trop. Med. Public Health.

[74] Waitayangkoon P., Thongkam A., Benjamungkalarak T., Rachayon M., Thongthaisin A., Chatsuwan T., Thammahong A., Chiewchengchol D. (2020). Hospital Epidemiology and Antimicrobial Susceptibility of Isolated Methicillin-Resistant Staphylococcus Aureus: A One-Year Retrospective Study at a Tertiary Care Center in Thailand. Pathog. Glob. Health.

[75] Son N.T., Huong V.T.T., Lien V.T.K., Nga D.T.Q., Au T.T.H., Hang P.T.T., Minh H.T.N., Binh T.Q. (2020). Antimicrobial Resistance Profile and Molecular Characteristics of Staphylococcus Aureus Isolates from Hospitalized Adults in Three Regions of Vietnam. Jpn. J. Infect. Dis..

[76] Chen C.J., Yang Lauderdale T.L., Huang Y.C. (2021). Evolution and Population Structures of Prevalent Methicillin-Resistant Staphylococcus Aureus in Taiwan. Front. Microbiol..

[77] Tang C.T., Nguyen D.T., Hoa N.T., Nguyen T.M.P., Le V.T., To S.D., Lindsay J., Nguyen T.D., Bach V.C., Le Q.T. (2007). An Outbreak of Severe Infections with Community-Acquired MRSA Carrying the Panton-Valentine Leukocidin Following Vaccination. PLoS ONE.

[78] Huang Y.C., Chen C.J. (2011). Community-Associated Meticillin-Resistant Staphylococcus Aureus in Children in Taiwan, 2000s. Int. J. Antimicrob. Agents.

[79] Yang X., Liu Y., Wang L., Qian S., Yao K., Dong F., Song W., Xu H., Zhen J., Zhou W. (2019). Clonal and Drug Resistance Dynamics of Methicillin-resistant Staphylococcus Aureus in Pediatric Populations in China. Pediatric Investig..

[80] Lawung R., Chuong L.V., Cherdtrakulkiat R., Srisarin A., Prachayasittikul V. (2014). Revelation of Staphylococcal Cassette Chromosome Mec Types in Methicillin-Resistant Staphylococcus Aureus Isolates from Thailand and Vietnam. J. Microbiol. Methods.

[81] Son N.T., Huong V.T.T., Lien V.T.K., Nga D.T.Q., Hai Au T.T., Nga T.T., Minh Hoa L.N., Binh T.Q. (2019). First Report on Multidrug-Resistant Methicillin-Resistant Staphylococcus Aureus Isolates in Children Admitted to Tertiary Hospitals in Vietnam S. J. Microbiol. Biotechnol..

[82] Van Nguyen K., Zhang T., Vu B.N.T., Dao T.T., Tran T.K., Nguyen D.N.T., Tran H.K.T., Nguyen C.K.T., Fox A., Horby P. (2014). Staphylococcus Aureus Nasopharyngeal Carriage in Rural and Urban Northern Vietnam. Trans. R. Soc. Trop. Med. Hyg..

[83] Vu B.N.T., Jafari A.J., Aardema M., Tran H.K.T., Nguyen D.N.T., Dao T.T., Nguyen T.V., Tran T.K., Nguyen C.K.T., Fox A. (2016). Population Structure of Colonizing and Invasive Staphylococcus Aureus Strains in Northern Vietnam. J. Med. Microbiol..

[84] Oliveira D.C., Tomasz A., de Lencastre H. (2001). The Evolution of Pandemic Clones of Methicillin-Resistant Staphylococcus Aureus: Identification of Two Ancestral Genetic Backgrounds and the Associated Mec Elements. Microb. Drug Resist..

[85] Thuy D.B., Campbell J., Hoang N.V.M., Trinh T.T.T., Duong H.T.H., Hieu N.C., Duy N.H.A., Van Hao N., Baker S., Thwaites G.E. (2017). A One-Year Prospective Study of Colonization with Antimicrobial-Resistant Organisms on Admission to a Vietnamese Intensive Care Unit. PLoS ONE.

[86] Thuy D.B., Campbell J., Hoang Nhat L.T., VanMinh Hoang N., Van Hao N., Baker S., Geskus R.B., Thwaites G.E., Van Vinh Chau N., Louise Thwaites C. (2018). Hospital-Acquired Colonization and Infections in a Vietnamese Intensive Care Unit. PLoS ONE.

[87] Thu T.A., Rahman M., Coffin S., Harun-Or-Rashid M., Sakamoto J., Hung N.V. (2012). Antibiotic Use in Vietnamese Hospitals: A Multicenter Point-Prevalence Study. Am. J. Infect. Control.

[88] Fishbain J.T., Viscount H.B. (2002). Surveillance for Methicillin-Resistant Staphylococcus Aureus in Battambang, Cambodia. Hawaii Med. J..

[89] Reed T.A.N., Krang S., Miliya T., Townell N., Letchford J., Bun S., Sar B., Osbjer K., Seng S., Chou M. (2019). Antimicrobial Resistance in Cambodia: A Review. Int. J. Infect. Dis..

[90] Vlieghe E., Sary S., Lim K., Sivuthy C., Phe T., Parry C., De Smet B., Monidarin C., Baron E., Moore C.E. (2013). First National Workshop on Antibiotic Resistance in Cambodia: Phnom Penh, Cambodia, 16–18 November 2011. J. Glob. Antimicrob. Resist..

[91] Chheng K., Tarquinio S., Wuthiekanun V., Sin L., Thaipadungpanit J., Amornchai P., Chanpheaktra N., Tumapa S., Putchhat H., Day N.P.J. (2009). Emergence of Community-Associated Methicillin-Resistant Staphylococcus Aureus Associated with Pediatric in Infection in Cambodia. PLoS ONE.

[92] Hirakawa S., Mori T., Ohkoshi Y., Aung M.S., Kobayashi N. (2022). An Infant Case of Cervical Purulent Lymphadenitis Caused by ST834 Community-Acquired Methicillin-Resistant Staphylococcus Aureus with SCCmec-IVc in Japan. New Microbes New Infect..

[93] Uehara Y., Sasaki T., Baba T., Lu Y., Imajo E., Sato Y., Tanno S., Furuichi M., Kawada M., Hiramatsu K. (2019). Regional Outbreak of Community-Associated Methicillin-Resistant Staphylococcus Aureus ST834 in Japanese Children. BMC Infect. Dis..

[94] Rao Q., Shang W., Hu X., Rao X. (2015). 2015 Staphylococcus Aureus ST121: A Globally Disseminated Hypervirulent Clone. J. Med. Microbiol..

[95] Nickerson E.K., Wuthiekanun V., Kumar V., Amornchai P., Wongdeethai N., Chheng K., Chantratita N., Putchhat H., Thaipadungpanit J., Day N.P. (2011). Emergence of Community-Associated Methicillin-Resistant Staphylococcus Aureus Carriage in Children in Cambodia. Am. J. Trop. Med. Hyg..

[96] Fox-Lewis A., Takata J., Miliya T., Lubell Y., Soeng S., Sar P., Rith K., McKellar G., Wuthiekanun V., McGonagle E. (2018). Antimicrobial Resistance in Invasive Bacterial Infections in Hospitalized Children, Cambodia, 2007–2016. Emerg. Infect. Dis..

[97] Phetsouvanh R., Phongmany S., Soukaloun D., Rasachak B., Soukhaseum V., Soukhaseum S., Frichithavong K., Khounnorath S., Pengdee B., Phiasakha K. (2006). Causes of Community-Acquired Bacteremia and Patterns of Antimicrobial Resistance in Vientiane, Laos. Am. J. Trop. Med. Hyg..

[98] Anderson M., Luangxay K., Sisouk K., Vorlasan L., Soumphonphakdy B., Sengmouang V., Chansamouth V., Phommasone K., Van Dyke R., Chong E. (2014). Epidemiology of Bacteremia in Young Hospitalized Infants in Vientiane, Laos, 2000–2011. J. Trop. Pediatrics.

[99] Kakinohana S., Uemura E., Insisiengmay S., Higa N., Iwanaga M. (2002). Staphylococcus Aureus Isolated from Hospital Staff: A Comparative Study of Laos and Japan. J. Infect. Chemother..

[100] Higa N., Sithivong N., Phantouamath B., Insisiengmay S., Miyazato T., Iwanaga M. (2004). Initial Stage of Hospital Contamination with Methicillin-Resistant Staphylococcus Aureus in Lao People’s Democratic Republic. J. Hosp. Infect..

[101] Yeap A.D., Woods K., Dance D.A.B., Pichon B., Rattanavong S., Davong V., Phetsouvanh R., Newton P.N., Shetty N., Kearns A.M. (2017). Molecular Epidemiology of Staphylococcus Aureus Skin and Soft Tissue Infections in the Lao People’s Democratic Republic. Am. J. Trop. Med. Hyg..

[102] Aung M.S., Urushibara N., Kawaguchiya M., Aung T.S., Mya S., San T., Nwe K.M., Kobayashi N. (2011). Virulence Factors and Genetic Characteristics of Methicillin-Resistant and -Susceptible Staphylococcus Aureus Isolates in Myanmar. Microb. Drug Resist..

[103] Aung M.S., San T., Urushibara N., San N., Oo W.M., Soe P.E., Kyaw Y., Ko P.M., Thu P.P., Hlaing M.S. (2020). Molecular Characterization of Methicillin-Susceptible and-Resistant Staphylococcus Aureus Harboring Panton-Valentine Leukocidin-Encoding Bacteriophages in a Tertiary Care Hospital in Myanmar. Microb. Drug Resist..

[104] Steinig E.J., Duchene S., Robinson D.A., Monecke S., Yokoyama M., Laabei M., Slickers P., Andersson P., Williamson D., Kearns A. (2019). Evolution and Global Transmission of a Multidrug-Resistant, Community-Associated Methicillin-Resistant Staphylococcus Aureus Lineage from the Indian Subcontinent. mBio.

[105] Aung M.S., Zi H., Nwe K.M., Maw W.W., Aung M.T., Min W.W., Nyein N., Kawaguchiya M., Urushibara N., Sumi A. (2016). Drug Resistance and Genetic Characteristics of Clinical Isolates of Staphylococci in Myanmar: High Prevalence of PVL among Methicillin-Susceptible Staphylococcus Aureus Belonging to Various Sequence Types. New Microbes New Infect..

[106] Aung M.S., San T., Urushibara N., San N., Hlaing M.S., Soe P.E., Htut W.H.W., Moe I., Mon W.L.Y., Chan Z.C.N. (2022). Clonal Diversity and Molecular Characteristics of Methicillin-Susceptible and -Resistant Staphylococcus Aureus from Pediatric Patients in Myanmar. Microb. Drug Resist..

[107] Sarkhoo E., Udo E.E., Boswihi S.S., Monecke S., Mueller E., Ehricht R. (2021). The Dissemination and Molecular Characterization of Clonal Complex 361 (CC361) Methicillin-Resistant Staphylococcus Aureus (MRSA) in Kuwait Hospitals. Front. Microbiol..

[108] Soe P.E., Han W.W., Sagili K.D., Satyanarayana S., Shrestha P., Htoon T.T., Tin H.H. (2021). High Prevalence of Methicillin-Resistant Staphylococcus Aureus among Healthcare Facilities and Its Related Factors in Myanmar (2018–2019). Trop. Med. Infect. Dis..

[109] Navarro-almario E.E., Velmonte M.A., Saniel M.C., Cruda C.L. (1988). An Evaluation of Recommended Heteroresistant Staphylococcus Aureus Tests for Detecting. Phil. J. Microbiol. Infect. Dis..

[110] We M., Torres T., Saniel M., Cruda-Pineda C., Cordero C., Antonio-Velmonte M. (1999). Nosocomial Acquisition of Oxacillin—Resistant Staphylococcus Aureus (ORSA) at the Philippine General Hospital. Philipp. J. Microbiol. Infect. Dis..

[111] Ontengo D., AL B., SR S., Ronald M., Tuazon O.A. (2004). Methicillin Resistant Staphylococcus Aureus Isolates from Filipino Patients 1999–2003. Phil. J. Microbiol. Infect. Dis..

[112] Juayang A.C., De Los Reyes G.B., De La Rama A.J.G., Gallega C.T. (2014). Antibiotic Resistance Profiling of Staphylococcus Aureus Isolated from Clinical Specimens in a Tertiary Hospital from 2010 to 2012. Interdiscip. Perspect. Infect. Dis..

[113] Valle D.L., Paclibare P.A.P., Cabrera E.C., Rivera W.L. (2016). Molecular and Phenotypic Characterization of Methicillin-Resistant Staphylococcus Aureus Isolates from a Tertiary Hospital in the Philippines. Trop. Med. Health.

[114] Breurec S., Zriouil S.B., Fall C., Boisier P., Brisse S., Djibo S., Etienne J., Fonkoua M.C., Perrier-Gros-Claude J.D., Pouillot R. (2011). Epidemiology of Methicillin-Resistant Staphylococcus Aureus Lineages in Five Major African Towns: Emergence and Spread of Atypical Clones. Clin. Microbiol. Infect..

[115] Buenaventura-Alcazaren F.A., dela Tonga A., Ong-Lim A., Destura R.V. (2020). Prevalence and Molecular Characteristics of MRSA Nasal Carriage among Hospital Care Workers in a Tertiary Hospital in the Philippines. J. Microbiol. Immunol. Infect..

[116] Ko J., Chung D.R., Park S.Y., Baek J.Y., Kim S.H., Kang C.I., Peck K.R., Lee N.Y., Song J.H. (2013). First Imported Case of Skin Infection Caused by PVL-Positive ST30 Community-Associated Methicillin-Resistant Staphylococcus Aureus Clone in a Returning Korean Traveler from the Philippines. J. Korean Med. Sci..

[117] Bochet M., Francois P., Longtin Y., Gaide O., Renzi G., Harbarth S. (2008). Community-Acquired Methicillin-Resistant Staphylococcus Aureus Infections in Two Scuba Divers Returning from the Philippines. J. Travel Med..

[118] Rubin I.M., Hansen T.A., Klingenberg A.M., Petersen A.M., Worning P., Westh H., Bartels M.D. (2018). A Sporadic Four-Year Hospital Outbreak of a ST97-IVa MRSA With Half of the Patients First Identified in the Community. Front. Microbiol..

[119] Sato T., Usui M., Motoya T., Sugiyama T., Tamura Y. (2015). Characterisation of Meticillin-Resistant Staphylococcus Aureus ST97 and ST5 Isolated from Pigs in Japan. J. Glob. Antimicrob. Resist..

[120] Wurster J.I., Bispo P.J.M., Van Tyne D., Cadorette J.J., Boody R., Gilmore M.S. (2018). Staphylococcus Aureus from Ocular and Otolaryngology Infections Are Frequently Resistant to Clinically Important Antibiotics and Are Associated with Lineages of Community and Hospital Origins. PLoS ONE.

[121] Masim M.L., Argimón S., Espiritu H.O., Magbanua M.A., Lagrada M.L., Olorosa A.M., Cohen V., Gayeta J.M., Jeffrey B., Abudahab K. (2020). Genomic Surveillance of Methicillin-Resistant Staphylococcus Aureus in the Philippines from 2013–2014. bioRxiv.

[122] Lestari E.S., Severin J.A., Filius P.M.G., Kuntaman K., Duerink D.O., Hadi U., Wahjono H., Verbrugh H.A. (2007). Antimicrobial Resistance among Commensal Isolates of Escherichia Coli and Staphylococcus Aureus in the Indonesian Population inside and Outside Hospitals. Eur. J. Clin. Microbiol. Infect. Dis..

[123] Severin J.A., Lestari E.S., Kuntaman K., Melles D.C., Pastink M., Peeters J.K., Snijders S.V., Hadi U., Duerink D.O., Van Belkum A. (2008). Unusually High Prevalence of Panton-Valentine Leukocidin Genes among Methicillin-Sensitive Staphylococcus Aureus Strains Carried in the Indonesian Population. J. Clin. Microbiol..

[124] Santosaningsih D., Santoso S., Budayanti N.S., Kuntaman K., Lestari E.S., Farida H., Hapsari R., Hadi P., Winarto W., Milheiriço C. (2014). Epidemiology of Staphylococcus Aureus Harboring the MecA or Panton-Valentine Leukocidin Genes in Hospitals in Java and Bali, Indonesia. Am. J. Trop. Med. Hyg..

[125] Gayatri A.A.A.Y., Utama S., Somia A., Merati T.P. (2015). MRSA Infection in Patients Hospitalized at Sanglah Hospital: A Case Series. Acta Med. Indones..

[126] Santosaningsih D., Erikawati D., Hakim I.A., Santoso S., Hidayat M., Suwenda A.H., Puspitasari V., Irhamni I., Kuntaman K., van Arkel A.L.E. (2019). Reducing Transmission of Methicillin-Resistant Staphylococcus Aureus in a Surgical Ward of a Resource-Limited Hospital in Indonesia: An Intervention Study. Infect. Prev. Pract..

[127] Santosaningsih D., Santoso S., Budayanti N.S., Suata K., Lestari E.S., Wahjono H., Djamal A., Kuntaman K., van Belkum A., Laurens M. (2016). Characterisation of Clinical Staphylococcus Aureus Isolates Harbouring MecA or Panton-Valentine Leukocidin Genes from Four Tertiary Care Hospitals in Indonesia. Trop. Med. Int. Health.

[128] Deurenberg R.H., Beisser P.S., Visschers M.J., Driessen C., Stobberingh E.E. (2010). Molecular Typing of Methicillin-Susceptible Staphylococcus Aureus Isolates Collected in the Yogyakarta Area in Indonesia, 2006. Clin. Microbiol. Infect..

[129] Sunagar R., Hegde N.R., Archana G.J., Sinha A.Y., Nagamani K., Isloor S. (2016). Prevalence and Genotype Distribution of Methicillin-Resistant Staphylococcus Aureus (MRSA) in India. J. Glob. Antimicrob. Resist..

[130] Santosaningsih D., Santoso S., Setijowati N., Rasyid H.A., Budayanti N.S., Suata K., Widhyatmoko D.B., Purwono P.B., Kuntaman K., Damayanti D. (2018). Prevalence and Characterisation of Staphylococcus Aureus Causing Community-Acquired Skin and Soft Tissue Infections on Java and Bali, Indonesia. Trop. Med. Int. Health.

[131] Gordon R.J., Lowy F.D. (2008). Pathogenesis of Methicillin-Resistant Staphylococcus Aureus Infection. Clin. Infect. Dis..

[132] Buntaran L., Hatta M., Sultan A.R., Dwiyanti R., Sabir M. (2013). Sccmec Type II Gene Is Common among Clinical Isolates of Methicillin-Resistant Staphylococcus Aureus in Jakarta, Indonesia. BMC Res. Notes.

[133] Kuntaman K., Hadi U., Setiawan F., Koendori E.B., Rusli M., Santosaningsih D., Severin J., Verbrugh H.A. (2016). Prevalence of Methicillin Resistant Staphylococcus Aureus from Nose and Throat of Patients on Admission to Medical Wards of Dr Soetomo Hospital, Surabaya, Indonesia. Southeast Asian J. Trop. Med. Public Health.

[134] Nelwan E.J., Sinto R., Subekti D., Adiwinata R., Waslia L., Loho T., Safari D., Widodo D. (2018). Screening of Methicillin-Resistant Staphylococcus Aureus Nasal Colonization among Elective Surgery Patients in Referral Hospital in Indonesia. BMC Res. Notes.

[135] Satyavani M., Momin J., Yapp S.K.S. (2012). Nosocomial Infections Surveillance in RIPAS Hospital. Brunei Int. Med. J..

[136] Abdul Malik N.F., Muharram S.H., Abiola O. (2014). Staphylococcus Aureus Nasal Carriage in Young Healthy Adults in Brunei Darussalam. Brunei Int. Med. J..

